# A randomized controlled clinical trial of cardiac telerehabilitation with a prolonged mobile care monitoring strategy after an acute coronary syndrome

**DOI:** 10.1002/clc.23757

**Published:** 2021-12-24

**Authors:** Ernesto Dalli Peydró, Nuria Sanz Sevilla, María T. Tuzón Segarra, Vicente Miró Palau, Jorge Sánchez Torrijos, Juan Cosín Sales

**Affiliations:** ^1^ Department of Cardiology Hospital Arnau de Vilanova Valencia Spain; ^2^ Department of Physical Medicine and Rehabilitation University Hospital Doctor Peset Valencia Spain; ^3^ Department of Cardiology University Hospital La Fe Valencia Spain

**Keywords:** cardiac rehabilitation, coronary heart disease, secondary prevention, telemedicine, telerehabilitation

## Abstract

**Background:**

Center‐based cardiac rehabilitation (CBCR) improves health outcomes but has some limitations. We designed and validated a telerehabilitation system to overcome these barriers.

**Methods:**

We included 67 low‐risk acute coronary syndrome patients in a randomized controlled trial allocated 1:1 to a 10‐month cardiac telerehabilitation (CTR) program or an 8‐week CBCR program. Patients underwent ergospirometry, blood tests, anthropometric measurements, IPAQ, PREDIMED, HADS, and EQ‐5D questionnaires at baseline and 10 months. Data collectors were blinded to the treatment groups.

**Results:**

The intention‐to‐treat analysis included 31 patients in the CTR group and 28 patients in the CBCR group. The primary outcome showed increased physical activity according to the IPAQ survey in the CTR group compared to the CBCR group (median increase 1726 METS‐min/week vs. 636, *p* = .045). Mean VO2max increased 1.62 ml/(kg min) (95% confidence interval [CI]: 0.56–2.69, *p* < .004) from baseline in the CTR group, and 0.60 mL/(kg min) (*p* = .40) in the CBCR group. Mean apoB/apoA‐I ratio decreased 0.13 (95% CI: −0.03 to 0.24, *p* = .017) in the CTR group, with no significant change in the CBCR group (*p* = .092). The median non‐HDL cholesterol increased by 7.3 mg/dl (IQR: −2.4 to 18.6, *p* = .021) in the CBCR group, but the increase was not significant in the CTR group (*p* = .080). Adherence to a Mediterranean diet, psychological distress, and quality of life showed greater improvement in the CTR group than in the CBCR group. Return‐to‐work time was reduced with the telerehabilitation strategy.

**Conclusion:**

This system allows minimal in‐hospital training and prolonged follow‐up. This strategy showed better results than CBCR.

## 1 INTRODUCTION

Cardiac rehabilitation is a multidisciplinary program recommended for patients with ST and non‐ST‐segment elevation acute coronary syndromes (ACS) and chronic coronary syndromes (class I, level of evidence A).^1^, ^2^, ^3^, ^4^ Despite the proven benefit, the attendance rate for these programs is 34%, according to the EUROSPIRE V registry.^5^ This situation has worsened as a result of the COVID‐19 pandemic.^6^ Cardiac telerehabilitation (CTR) has shown to be at least equally beneficial and cost‐effective than center‐based cardiac rehabilitation (CBCR).^7^, ^8^, ^9^

We developed a cardiac telerehabilitation system called *Cardioplan*, which consists of a web platform and a smartphone application that allows prolonged telemonitoring follow‐up after minimal patient training. To validate this approach, a clinical trial was designed to compare a 10‐month program of telerehabilitation with a conventional 8‐week program of hospital cardiac rehabilitation. In addition to testing a previously unevaluated strategy, this study was conducted during a period of lockdown and restricted social mobility due to the COVID‐19 pandemic and provides additional insight on what telerehabilitation can offer in this situation.

## 2 METHODS

### 2.1 Study design

This is a randomized controlled trial, with an intervention group that followed a CTR program in Hospital Arnau de Vilanova, and a control group that followed a CBCR program in University Hospital La Fe.

Patients with ACS willing to participate were randomly assigned (1:1) to either CTR or CBCR. Randomization was performed by an independent statistician. Seven blocks were randomly selected and the assignments were stored in seven packages of 10 closed envelopes. These packages were handed over to the principal investigator, who only opened them after recruitment of a new participant.

Each participant signed an informed consent form before participation and was notified of their group allocation after completing their baseline exercise test after 12 days of hospital discharge. Baseline measurements were done at this moment. All assessments were completed at the hospital Arnau de Vilanova. Patients were asked not to disclose their trial group to the faculty, including the investigators involved in questionnaire administration and cardiopulmonary stress testing (CPET) evaluations. Visits to primary care physicians and corresponding specialists were not impacted by the trial.

The trial was conducted according to the ethical principles of the Declaration of Helsinki. The study was approved by the ethics committee of Hospital Arnau de Vilanova and the Spanish Agency of Medicines and Medical Devices (484/14/EC) (Supporting Information Material 1). Each participant signed an informed consent form prior to before participation. This report follows the CONSORT‐EHEALTH guidelines (Supporting Information Material 2).^10^

### 2.2 Study population

Patients were recruited by face‐to‐face assessment at discharge after ACS between May 28, 2019 and March 10, 2020, when recruitment was stopped due to the COVID‐19 pandemic. Patient age was limited to 18–72 years old. All included patients had to meet low‐risk criteria, left ventricular ejection fraction ≥ 50%, and have minimum smartphone usage skills. The main exclusion criteria were reduced mobility, pulmonary diseases, neoplasms, or cognitive impairment.

### 2.3 Control and experimental rehabilitation procedures

Both groups were given the same education. The target heart rate during exercise sessions was 60%–80% of the heart rate reserve based on the baseline treadmill test. During follow‐up, patients were instructed to engage in recommended moderate physical activity guided by Borg's rating of perceived exertion scale of 12–14 (6–20 scale), as well as strength exercises twice a week. Warm‐up, stretching, and resistance‐band exercises were included in both groups.

### 2.4 Telerehabilitation group

A portion of hospital training, comprising 2 weeks with four supervised sessions of exercise, was completed. Physical activity consisted of walking down a corridor, adjusting their pace to attain a target heart rate as measured by their smartphone and heart rate monitor (Polar H7). The smartphone application guided participants through a daily exercise and data entry program for 10 months.

### 2.5 CBCR group

The CBCR program comprised 2 months of treatment with 16 sessions of supervised exercise. Physical activity consisted of routine workouts and aerobic cycling training.

### 2.6 Description of the comprehensive monitoring system

The system, designed in cooperation with Trilema Salud (Valencia, Spain), consists of the following elements:

1. A webpage that allowed personalized healthcare and tracking of patient adherence to recommendations with password‐protected access. The healthcare team monitored seven variables based on a traffic light color code and communicated with patients if necessary.
2. A smartphone application that allowed daily scheduling of exercise sessions; recording of subjective general condition, vital signs, and medication adherence; and gave a recommended diet. The exercise module tracked and recorded every exercise session and provided access to warm‐up and stretching videos, a virtual educational classroom, and suggested websites. Access was password‐protected to ensure confidentiality. Technical assistance in the case of sensor/system failure was provided through a call center.

### 2.7 Outcome measures

The primary outcome was an increase in self‐reported physical activity in MET‐min/week, derived from the IPAQ questionnaire. The main secondary outcome was an increase in the VO2max. Additional secondary outcomes included other CPET measurements, changes in laboratory parameters, anthropometric variables, adherence to the rehabilitation program, returning to work, adherence to a Mediterranean diet, psychological well‐being, health‐related quality of life, and smoking cessation.

### 2.8 Cardiopulmonary stress test

Symptom‐limited CPET was performed after hospital discharge and at 10 months. Stress testing was based on a Bruce protocol using an ergospirometer (Jaeger, MS‐CPX). Heart rate, blood pressure, 12‐lead ECG, and breath‐by‐breath respiratory gas analyses were recorded. The test was assumed to be maximal in case of a respiratory gas exchange ratio (RER) > 1.1 or cardiac heart rate >85% of the maximal predicted heart rate. VO_2_ was defined as the maximal oxygen uptake during the final 30 s of the test. The final CPET was performed with or without beta‐blockers, depending on their use at baseline.

### 2.9 Blood tests and lipid measurements

Blood samples were drawn at baseline and at 4 and 10 months follow‐up and were subsequently analyzed in the hospital laboratory for a standard panel. Medications other than lipid‐lowering therapy were allowed to be modified during the study period. If LDL cholesterol was above 100 mg/dl at 4 months, the treatment was modified, and the patient was excluded from the lipid substudy.

### 2.10 Body composition and waist circumference

Measurements at baseline, after 4 months of follow‐up, and after 10 months of follow‐up, including weight and visceral fat were assessed using a Tanita BC‐602 scale, Japan. Waist circumference was measured midway between the costal border and the iliac crest.

## 3 QUESTIONNAIRES

Questionnaires were administered at baseline and at 10 months through computer‐assisted face‐to‐face interviews to obtain higher response rates. The interviewer did not know the patient's rehabilitation group. The International Physical Activity Questionnaire (IPAQ) consists of seven questions about physical activity (intense, moderate, or walking) in the last 7 days, as well as the time spent sitting on a weekday. The MET‐minute/week is calculated by multiplying the value of the exercise level (3.3, 4, or 8) by the duration in minutes of the daily activity and by the number of days per week that it is performed. The level of physical exercise can be categorized as low, moderate, or high.^11^, ^12^

The Prevention with Mediterranean Diet (PREDIMED) questionnaire includes 14 items. A score of 9 or more reflects good adherence.^13^

The Hospital Anxiety and Depression Scale (HADS) consists of 14 items divided into anxiety and depression subscales. The reference period was the previous week.^14^

The EQ‐5D‐5L questionnaire consists of two parts. The first part measures five aspects of health. The second part is a scale from 0 (worst state of health) to 100 (best state of health).^15^

### 3.1 Sample size

Based on the potential impact of the intervention, a greater increase in total MET‐min/week derived from the IPAQ questionnaire in the experimental group was considered plausible. This gave rise to expected mean values 25% higher at the end of rehabilitation for the experimental group (5000 MET‐min/week vs. 4000 for the CBCR group) (*SD* in both groups was considered equal to 1000).^16^, ^17^ A total of 30 patients in each group was calculated to provide 95% power at the 5% level of significance to detect a statistically significant difference between groups using the Mann–Whitney *U* test. A 12% loss to follow‐up was estimated; therefore, 70 patients were planned to be enrolled in the study.

### 3.2 Statistical analysis

Outcomes were analyzed according to the intention‐to‐treat principle. To analyze treatment effects within groups (at 4 months or 10 months), we used the McNemar–Bowker test of symmetry for qualitative variables (McNemar test for dichotomous variables) or Student's *t‐test* for paired samples for quantitative variables (Wilcoxon signed‐rank test when parametric assumptions could not be assumed). The comparison of treatments between groups was carried out using Pearson *χ* ^2^ test for qualitative variables (Fisher exact test for dichotomous variable) or Student's *t‐test* for independent samples for quantitative variables (Mann–Whitney *U* test when parametric assumptions could not be assumed). The relationship between two variables was assessed by Pearson's correlation coefficient (Spearman's rank correlation coefficient when parametric assumptions could not be assumed). Two‐sided exact *p* values were calculated whenever possible, and *p* ≤ 0.05 were considered statistically significant. Data were analyzed using IBM SPSS Statistics 22 and R 4.0.2 for Microsoft Windows.

## 4 RESULTS

### 4.1 Patients and program adherence

**Figure 1:**
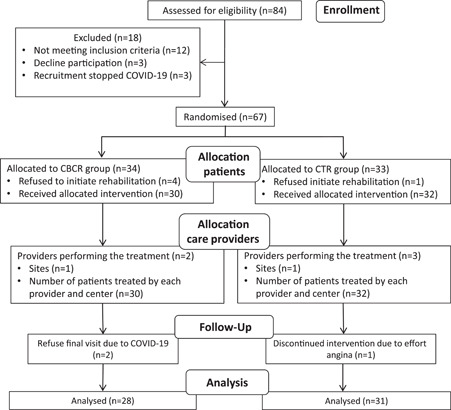
CONSORT patient flow diagram for nonpharmacologic treatment trials

A total of 67 patients were enrolled in the study, but only 59 were included in the intention‐to‐treat analysis: 31 and 28 were randomized to the CTR and CBCR groups, respectively (CONSORT Flow chart, Figure 1). There were no significant differences between the groups at baseline (Table 1).

**Table 1:** Baseline demographics, clinical characteristics, cardiopulmonary exercise testing parameters, and medication use

	CTR group (*n* = 31)	CBCR group (*n* = 28)	*p*
Demographic characteristics			
Age (years), mean (*SD*)	57.5 (9.0)	54.7 (9.9)	.266
Male, *n* (%)	27 (87.1%)	27 (96.4%)	.356
Weight (kg), mean (*SD*)	81.6 (15.9)	84.5 (13.0)	.447
Visceral fat (%), median (IQR)	12 (10–13)	12 (10–14)	.663
BMI (kg/m^2^), mean (*SD*)	27.0 (4.2)	27.7 (3.5)	.158
Waist circumference (cm), mean (*SD*)	98.4 (13.4)	101.3 (9.1)	.357
HbA1c (%), mean (*SD*)	5.89 (0.46)	5.72 (0.55)	.235
Total cholesterol (mg/dl), mean (*SD*)	127.1 (32.4)	120.6 (20.6)	.389
HDL cholesterol (mg/dl), mean (*SD*)	46.6 (12.1)	46.0 (10.1)	>.800
LDL cholesterol (mg/dl), mean (*SD*)	59.3 (27.1)	54.2 (16.8)	.565
Triglycerides (mg/dl), median (IQR)	91 (77–121)	84 (72–118)	.532
ApoB/apoA‐I ratio, mean (*SD*)	0.62 (0.28)	0.56 (0.20)	.381
LP(a) (mg/dl), median (IQR)	44 (11–89)	31 (17–64)	.388
IPAQ questionnaire			
Total MET‐min/week median (IQR)	1251 (693–2624)	1502 (896–3924)	.186
Cardiopulmonary exercise testing			
HRmax (bpm), mean (*SD*)	136.5 (18.7)	137.5 (20.1)	>.800
Exercise time (min), mean (*SD*)	7.14 (2.29)	7.69 (2.21)	.354
RERmax, mean (*SD*)	1.17 (0.08)	1.22 (0.10)	.049
VO2max (ml (kg^ ^min)), mean (SD)	23.94 (4.64)	23.68 (4.48)	>.800
Cardiovascular risk factors			
Total (number), median (IQR)	2 (1–2)	2 (1–3)	.500
Hypertension, *n* (%)	16 (51.6%)	13 (46.4%)	.796
Smoking, *n* (%)	14 (45.2%)	18 (64.3%)	.193
Dyslipidaemia, *n* (%)	14 (45.2%)	14 (50.0%)	.797
Diabetes mellitus, *n* (%)	6 (19.4%)	6 (21.4%)	>.800
Acute coronary syndrome			.763
UA, *n* (%)	9 (29.0%)	6 (21.4%)	
NSTEMI, *n* (%)	9 (29.0%)	10 (35.7%)	
STEMI, *n* (%)	13 (41.9%)	12 (42.9%)	
Medication			
Dual antiplatelet therapy, %	30 (96.8%)	27 (96.4%)	>.800
Statins, *n* (%)	31 (100.0%)	26 (92.9%)	.221
B‐blockers, %	20 (64.5%)	22 (78.6%)	.264
ACE inhibitors/ARBs, %	22 (71.0%)	20 (71.4%)	>.800
Systolic function			
LVEF (%), mean (*SD*)	62.6 (7.4)	62.0 (6.4)	.709
Coronary arteries			
Coronary lesions, median (IQR)	1 (1–2)	1 (1–1)	.225
Number of stents, median (IQR)	1 (1–2)	1 (1–1)	.222

Abbreviations: BMI, body mass index; HbA1c, glycosylated hemoglobin; IQR, interquartile range; LVEF, left ventricular ejection fraction; NSTEMI, non‐ST elevation myocardial infarction; RER, respiratory exchange ratio; STEMI, ST elevation myocardial infarction; UA, unstable angina; VO2max, maximal oxygen uptake.

Patients with at least one training session for both groups were included in the follow‐up period. Eight patients (12%) were excluded: six in the CBCR group and two in the CTR group. A total of seven women were randomized (10.4%), four in the CTR group and three in the CBCR. All women in the CTR group attended the sessions, but one declined follow‐up. In the CBCR group, two women did not start rehabilitation, and the third completed only three sessions. During the study period, three patients were readmitted. In the CBCR group, one patient with postinfarction angina received stenting in a new vessel. Two patients in the CTR group with unstable angina had known distal lesions in the stented vessel and were not amenable to revascularization. One patient was excluded due to refractory angina.

### 4.2 Primary outcome

**Figure 2:**
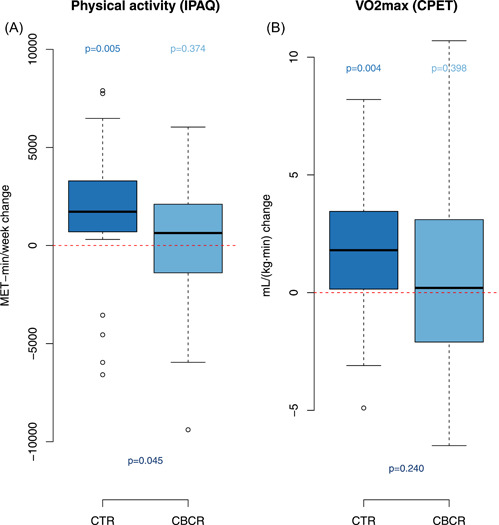
Changes in self‐reported physical activity through the International Physical Activity Questionnaire (IPAQ) (Panel A). Changes in maximal oxygen uptake (VO2max) during cardiopulmonary exercise testing (Panel B). CBCR, center‐based cardiac rehabilitation; CTR, cardiac telerehabilitation

The increase in total physical activity was significantly higher in the CTR group than in the CBCR group (median increase of 1726 METS‐min/week for CTR vs. 636 METS‐min/week for CBCR group, *p* = .045) (Figure 2). The correlation between MET‐min/week and VO2max before and after rehabilitation was *ρ* = 0.224 (*p* = .091) and 0.311 (*p* = .018), respectively.

### 4.3 Secondary outcomes

#### 4.3.1 Cardiopulmonary exercise testing

In the CTR group, VO2max mean increase from baseline was 1.62 ml/(kg min) (95% CI: 0.56–2.69, *p* = .004) and in the CBCR group it was 0.60 ml/(kg min) (95% CI: −0.83 to 2.03, *p* = .40) (Figure 2). The most relevant data can be observed in the Table 2.

**Table 2:** Physical activity through the International Physical Activity Questionnaire (IPAQ) and cardiopulmonary exercise test (CPET) parameters at baseline and after the 10‐month follow‐up period

	CTR group (*n* = 30)			CBCR group (*n* = 28)			
	Baseline	Final	p_1_	Baseline	Final	p_2_	p_12_
IPAQ							
Total MET‐min/week, *m* (IQR)	1251 (693–2624)	4031 (1875–5973)	0.005	1502 (896–3924)	2420 (1391–4997)	0.374	0.045
Walking, *m* (IQR)	743 (231–1386)	1287 (990–2079)	<0.001	644 (330–1386)	1172 (743–1386)	0.070	0.350
Moderate activity, *m* (IQR)	140 (0–720)	580 (240–2400)	0.033	440 (0–2700)	540 (0–1020)	0.548	0.039
Vigorous activity, *m* (IQR)	0 (0–320)	620 (0–2400)	0.053	0 (0–240)	0 (0–1960)	0.313	0.484
Hours a day sitting, *m* (IQR)	7 (5–8)	6 (4–7)	0.147	6 (5–9)	7 (6–8)	>0.800	0.215
Weekly energy expenditure, *m* (IQR)	1524 (773–3892)	5445 (2905–8772)	0.002	2207 (1334–8505)	3349 (1956–7491)	0.767	0.036
Effort level			<0.001			0.343	0.031^a^
Inactive/low, *n* (%)	6 (20.0%)	1 (3.3%)		6 (21.4%)	3 (10.7%)		
Moderate, *n* (%)	17 (56.7%)	6 (20.0%)		12 (42.9%)	13 (46.4%)		
High, *n* (%)	7 (23.3%)	23 (76.7%)		10 (35.7%)	12 (42.9%)		
CPET							
VO2max (ml/(kg min), mean (*SD*) (*n*_1_ = 31, *n*_2_ = 28)	23.9 (4.6)	25.6 (5.6)	0.004	23.7 (4.5)	24.3 (5.5)	0.398	0.240
VO2max (%), mean (*SD*) (*n*_1_ = 31, *n*_2_ = 28)	89.6 (12.2)	96.3 (15.5)	0.001	85.4 (15.5)	88.3 (17.8)	0.216	0.174
METS, mean (*SD*) (*n*_1_ = 31, *n*_2_ = 28)	6.76 (1.26)	7.29 (1.59)	0.001	6.88 (1.31)	6.98 (1.54)	0.698	0.131
VEmax (l/min), mean (*SD*) (*n*_1_ = 31, *n*_2_ = 28)	72.6 (18.0)	79.4 (20.6)	0.010	75.1 (21.9)	76.2 (22.1)	0.701	0.138
RERmax, mean (*SD*) (*n*_1_ = 31, *n*_2_ = 28)	1.17 (0.08)	1.20 (0.09)	0.091	1.22 (0.10)	1.18 (0.11)	0.131	0.030
Heart rate max (bpm), mean (*SD*) (*n*_1_ = 31, *n*_2_ = 28)	136.5 (18.7)	145.0 (17.2)	<0.001	137.5 (20.1)	141.0 (16.2)	0.186	0.130
Heart rate max (%), mean (*SD*) (*n*_1_ = 31, *n*_2_ = 28)	84.2 (10.4)	90.0 (9.6)	<0.001	83.6 (11.3)	85.4 (9.7)	0.192	0.035
Blood pressure max (mmHg), mean (*SD*) (*n*_1_ = 31, *n*_2_ = 28)	166.8 (21.7)	166.3 (27.8)	>0.800	169.6 (27.7)	161.3 (25.4)	0.124	0.301
Cardiac output max (l/min), mean (*SD*) (*n*_1_ = 31, *n*_2_ = 28)	11.9 (2.8)	12.6 (3.2)	0.010	12.3 (2.8)	12.8 (3.1)	0.128	0.779
BRmax (b/min), mean (*SD*) (*n*_1_ = 29, *n*_2_ = 26)	24.8 (19.0)	26.4 (19.5)	0.655	23.5 (19.8)	32.4 (16.7)	0.028	0.180
Effort time (min), mean (*SD*) (*n*_1_ = 31, *n*_2_ = 28)	7.14 (2.29)	8.59 (2.60)	<0.001	7.69 (2.21)	8.18 (2.32)	0.273	0.055
Treadmill speed (km/h), m(IQR) (*n*_1 _= 31, *n*_2_ = 28)	5.4 (4.0–6.6)	6.7 (5.4–6.7)	<0.001	5.4 (5.3–6.7)	5.5 (5.4–6.7)	0.154	0.019
Elevation (%), m(IQR) (*n*_1_ = 31, *n*_2_ = 28)	14 (12–16)	16 (12–18)	<0.001	14 (12–18)	14 (12–18)	0.218	0.006

Abbreviations: BR, breathing reserve; IQR, interquartile range; m, median; n1(n2), the sample size for the effect analysis in the CTR (CBCR) group; p1 (p2), change in the CTR (CBCR) group; p12, comparison of changes between the two groups; RER, respiratory exchange ratio; VEmax, maximal ventilation; VO2max (%), percentage of theoretical maximal oxygen uptake; VO2max, maximal oxygen uptake.

## 5 QUESTIONNAIRES

From the IPAQ questionnaire, the percentage of patients who reported a high level of effort at the end of the study period was significantly higher in the CTR group than in the CBCR group (76.7% vs. 42.9%, *p* = .031) (Table 2).

The HADS global score improved significantly from baseline in both groups, but the improvement was significantly greater in the CTR group than in the CBCR group (*p* = .015). The anxiety subscale showed a significantly greater effect in the CTR group (*p* = .006), whereas the depression subscale only improved significantly in the CTR group (*p* = .020).

The PREDIMED score improved significantly from baseline in both groups, with no differences between groups (*p* = .345). The percentage of patients reporting high adherence to the Mediterranean diet (score > 9 points) was higher in the CTR group (70%) than in the CBCR group (32%) (*p* = .001).

The global EQ‐5D‐5L questionnaire index only increased significantly from baseline in the CTR group, with no differences between groups (*p* = .261). The self‐assessment of health improved in both groups but was only significant in the CTR group (*p* = .008) (See Supporting Information Material 3).

### 5.1 Blood test parameters

**Figure 3:**
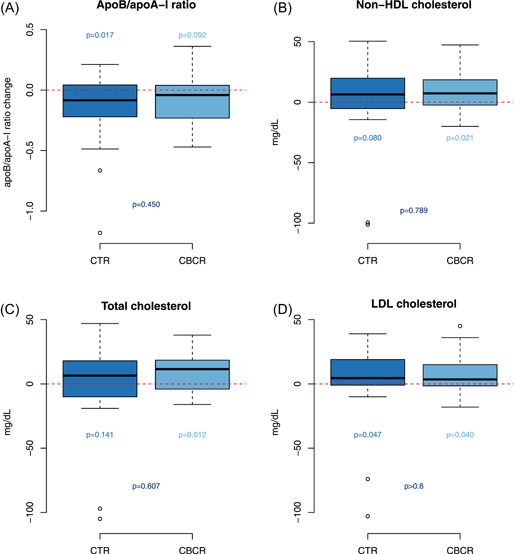
Effect of prolonged cardiac telerehabilitation (CTR) and center‐based cardiac rehabilitation (CBCR) on apoB/apoA‐I ratio, non‐HDL cholesterol, total cholesterol, and LDL cholesterol at the 10‐month follow‐up

ApoB/apoA‐I ratio decreased by 0.13 (95% CI: −0.03 to 0.24, *p* = .017) in the CTR group and by 0.08 (95% CI: −0.01 to 0.17, *p* = .092) in the CBCR group. Non‐HDL cholesterol increased a median of 7.3 mg/dL (IQR: −2.4 to 18.6, *p* = .021) in the CBCR group and by 2.1 mg/dl (IQR: −5.3 to 19.8, *p* = .080) in the CTR group. Total cholesterol increased by 11.5 mg/dl (IQR: −4 to 18.5, *p* = .012) in the CBCR group and 6.5 mg/dl (IQR: −10 to 18, *p* = .141) in the CTR group. No significant differences in lipid parameters were found between the groups (Figure 3). Two patients in the CTR group improve lipid control after starting the rehabilitation program, not from hospital discharge. One patient in the CBCR group was excluded at 4 months because of an LDL level >100 g/dl (see all blood test results in Supporting Information Material 4).

### 5.2 Anthropometric measurements

At 4 months, weight change in the CTR group was −2.24 kg (95% CI: −0.09 to 4.38, *p* = .042) and −0.64 kg (95% CI: −1.28 to 2.55, *p* = .495) in the CBCR group. At 10 months, weight change in the CTR group was −0.22 Kg (95% CI: −1.86 to 2.30, *p* > .8) and −0.1% in visceral fat from baseline, while the weight change in the CBCR group was 1.29 kg (95% CI: −0.44 to 3.02, *p* = .099) and +0.5% in visceral fat (*p* = .11). No intergroup differences were observed.

### 5.3 Smoking

Smoking cessation was observed in 50% of the previous smokers in both groups.

#### 5.3.1 Interval time to start the program and returning to work

Delay to first rehabilitation session from discharge was 51.9 ± 24.5 days in the CTR group and 72.5 ± 25.7 days in the CBCR group (*p* < .002) based solely on being able to access care. Returning to work took 113.6 ± 100.6 days in the CTR group and 245.7 ± 172 days in the CBCR group (*p* < .013).

#### 5.3.2 Users' experience with the cardiac rehabilitation app

The app user experience was assessed using the system usability scale. The overall score was 80.4 out of 100. Requested data was provided at rates of 47% for exercise sessions, 59% for food intake, and 54% for treatment validation. Data entry was considered poor (less than 20%) in seven patients. The users' main complaints were internet connection problems and handling difficulties in older patients.

## 6 DISCUSSION

Our trial showed that a 10‐month program of CTR, the longest reported intervention, increased physical activity, and oxygen consumption, improved the lipid risk profile, quality of life, and encouraged adherence to the rehabilitation program, compared to a CBCR program.

The primary outcome of our CTR participants showed a significant increase in self‐reported physical activity. This result might be related to the longer duration or higher adherence to the program. In a recent statement on home‐based cardiac rehabilitation of the AACVPR/AHA/ACC, Thomas et al. concluded that in at least 20 of the studies reviewed, the effect of CTR on exercise capacity improvement (i.e., peak oxygen uptake) appears to be similar to that observed with CBCR when programs last the same length of time.^18^ However, additional 6 months of telerehabilitation after a CBCR period increased oxygen consumption and self‐reported physical activity on the IPAQ survey, compared to CBCR alone.^17^ Similarly, our first secondary outcome demonstrated an increase in VO2max from baseline only in the CTR group. In this trial, the app contributed to self‐management and self‐care.

The decrease in the apoB/apoA‐I ratio in the CTR group and the significant increase in non‐HDL cholesterol in the CBCR group is of particular significance. In the INTERHEART study, the apoB/apoA‐I ratio was one of the main predictors of acute myocardial infarction and it was the best predictor of coronary events in the IDEAL study.^19^, ^20^ In patients treated with statins, elevated apoB and non‐HDL cholesterol are associated with the residual risk of all‐cause mortality and myocardial infarction.^21^ The stability of the lipid profile in the CTR group achieved by a nonpharmacological approach could be attributed to increased physical activity, nutritional improvement, or reminders to take medication delivered by the app.

The lower participation of women in cardiac rehabilitation programs remains a cause for concern as lower adherence leads to higher mortality.^18^, ^22^, ^23^, ^24^ In this study, a reduced delay from discharge to initiating the program in the CTR group could have influenced their higher adherence rates. The longest time to starting rehabilitation in the CBCR group was partly due to unforeseen delays in some patients in the scheduled visit to a rehabilitation physician by staff unavailability. It is possible that this may have led to higher dropout rates in the CBCR group.

Conclusions cannot be drawn from such a small number of participants, so further studies should be conducted to confirm if the higher drop‐out rate in CBCR can be mitigated using a CTR strategy, especially for women.

It has been observed that 67%–93% of patients return to work 2–3 months after ACS, but this probability decreases for patients aged over 55 years and in women. We found a significant reduction in the return‐to‐work period in the CTR group. This difference could be attributed to a self‐confidence effect, as previously suggested by Reibis et al.^25^ These data relating to returning to work are also in agreement with the results observed in the FIT@Home study.^26^

The minimal decrease in weight and visceral fat in the CTR group, contrasted with the nearly significant 1.3 kg gain and 0.5% visceral fat increase in the CBCR group. The weight gain in the CBCR group was similar to the reported 1–3 kg gain for the general Spanish population during the lockdown period, which took place from March 15 to June 21, 2020.^27^

Frequent questioning about intake of recommended foods could have exerted a motivational effect to improve adherence to the Mediterranean diet in the CTR group, as demonstrated by the PREDIMED questionnaire.

Several surveys during the COVID‐19 pandemic have suggested a higher prevalence of anxiety, depression, and lower well‐being compared to historical estimates.^28^ Experiencing an ACS could have worsened fear and uncertainty in our patients. The benefit observed in the CTR group, especially in the EQ‐5D index score, and depression scale (HADS), was noticeable. Long‐term monitoring appeared to have a positive effect on behavioral change and self‐confidence level and could have contributed to participants' earlier return to their social and occupational circles, which would merit confirmation in future studies.

Our findings in this field represent a step forward given the superiority of this extended follow‐up strategy, which was also noted in a previous study.^17^ Our results show it may be a more effective alternative to conventional rehabilitation, at least in low‐risk patients. This new scheme could achieve the hypothetical objective of an 80% inclusion rate of eligible patients, enable an early return to daily life, and make efficient use of health resources.

## 7 CONCLUSIONS

This study shows that a comprehensive telemonitoring system, with minimal hospital training and follow‐up of at least 10 months, increases physical activity and oxygen consumption; improves the quality of life, emotional well‐being, and adherence to the Mediterranean diet; and ameliorates lipid profile. The program also reduces dropouts and favors the return to work. Therefore, telerehabilitation overcame some barriers of traditional hospital rehabilitation, especially during a pandemic situation. The limited number of subjects prevents the results from being broadly applicable, although they do encourage further multicentre studies.

## CONFLICT OF INTERESTS

The authors declare that there are no conflict of interests.

## PREVIOUS PRESENTATIONS

The content of this article has not been previously presented.

## Supporting information

Supporting information.

Supporting information.

Supporting information.

Supporting information.

## Data Availability

The data that support the findings of this study are available from the corresponding author upon reasonable request.
