# Supplementary material for: A randomized controlled clinical trial of cardiac telerehabilitation with a prolonged mobile care monitoring strategy after an acute coronary syndrome
Source: Clin Cardiol. 2021 Dec 24;45(1):31–41. doi: 10.1002/clc.23757 (PMC8799046; doi:10.1002/clc.23757)
Supplement: Supplementary file 1 — Supporting information. [file CLC-45-31-s001.docx]

**RESEARCH PROTOCOL**

| Title | **Prevention and rehabilitation after acute coronary syndrome: new strategy through telemonitoring** |
| --- | --- |
| Promotor | Catcronic SL ( Nabelia)  Ronda Isaac Peral 9  Parque tecnológico de Paterna  46980 Valencia. Spain |
| Lead Researcher | Dr. Ernesto Dalli Peydró  Cardiology Service  Hospital Arnau de Vilanova  C/ San Clemente 12  46015 Valencia |
| Protocol Code | TSB2014 |
| 5^th^ Version | March 30 2020 |

Confidential Information

The information contained in this document is the promotor’s property, therefore, it is provided to you on a confidential bases for you, your research team, the Clinical Research Ethics Committee and the competent authorities, to use it. This information will not be revealed to other people without the previous written authorization of the promotor.

CONTACT DATA

Lead Researcher Dr. Ernesto Dalli Peydró

Cardiology Service

Hospital Arnau de Vilanova

C/ San Clemente 12

46015 Valencia

Phone: +34 961976190

FAX: +34 961976190

e-mail: [dalli@ono.com](mailto:dalli@ono.com)

Promotor Catcronic Salut, SL. (Nabelia)

Clinical trial management Vicente Pellicer Iborra

Ronda Isaac Peral 9

Parque tecnológico de Paterna

46980 Valencia. Spain

Phone 609801300

e-mail: [vicente.pellicer@nabelia.es](mailto:vicente.pellicer@nabelia.es)

Monitoring Juan Carlos Carrillo Gómez

Ronda Isaac Peral 9

Parque Tecnológico de Paterna

Valencia. Spain

Phone 695495662

e-mail: juancarlos.carrillo@nabelia.es

**LEAD RESEARCHER’S SIGN OF THE PROTOCOL PAGE**

**Prevention and rehabilitation after acute coronary syndrome: new strategy trough telemonitoring**

I have read the protocol and I agree that it contains all the necessary details in order to perform this study. I will conduct this study in accordance with the protocol and the Buena Práctica Clínica guidelines. I will provide copies of the protocol and all the pharmacological information related to the clinical and preclinical experience which has been provided to me and all the doctors who participate in this study. I will discuss this material with them to assure they all fully informed about the drugs under study and the conduct of the study. I agree to preserve all the patient’s registering information (Data Collection Booklets and Declaration of Informed Consent by patients) and any other information obtained in this study during a minimum period of time of 15 years.

_____________________________________

Lead Researcher’s Name

__________________________ __________

Lead Researcher’s Signature Date

# Summary

## Address and Protocol Identification

Catcronic Salut SL. (Nabelia)

Ronda Isaac Peral 9.

Parque Tecnológico de Valencia

46980 Paterna, Valencia.

## Study Title

Prevention and rehabilitation after acute coronary syndrome: new strategy trough telemonitoring.

## Protocol Code

TSB2014

## Lead Researcher

Dr. Ernesto Dalli Peydró

Cardiology Service

Hospital Arnau de Vilanova.

c/ San clemente 12

46015 Valencia

Phone +34 961976190

FAX +34 961976190

Email. [dalli@ono.com](mailto:dalli@ono.com)

## Centres where the study is planned to be carried out

Hospital Universitario Arnau de Vilanova. Valencia.

## CEICs that evaluate it

Hospital Universitario Arnau de Vilanova, Valencia.

## General Objective

To demonstrate that a telerehabilitation program improves physical activity compliance at 10 months follow-up, relative to a conventional rehabilitation program.

## Secondary Objectives

Very the improvement of the following parameters, after the intervention:

- Demonstrate an increase in oxygen consumption (VO2max).

- Demonstrate increased exertion time.

- Demonstrate an increase in workload.

- Decrease the presence of symptoms.

- Obtain improvement in lipid profile

- Decrease in ultrasensitive C Reactive Protein (CRPus)EQ

- Demonstrate increased adherence to the Mediterranean diet.

- Demonstrate improved results in psychological state.

- Demonstrate increased willingness to quit smoking and smoking cessation.

- Demonstrate a decrease in the sedentary lifestyle indicator.

- Improved measurement of carotid-femoral pulse wave velocity.

- To evaluate the cost per patient/month in the control cases of conventional rehabilitation and extended tele-rehabilitation intervention.

- To evaluate the time to return to work.

## Design

Randomised, controlled, clinical trial with two arms (cardiac tele-rehabilitation group and conventional in-hospital rehabilitation group).

## Disease or disorder under study

Acute Coronary Syndrome

## Drug Data

Does not apply.

## Population under study and total number of subjects

The study population consists of men and women with a maximum age of 72 years diagnosed with acute coronary syndrome who will be enrolled in a cardiac rehabilitation program. Sixty patients will be included in two groups of 30 subjects assigned to each rehabilitation program.

## Inclusion Criteria

- Patients after uncomplicated acute coronary syndrome of both sexes.

- Completion of a maximal or symptom-limited exercise test without angina or electrical ischaemia.

- Age 72 years or less.

## Exclusion Criteria

- Refusal of informed consent

- At the discretion of the investigator, primarily due to advanced biological age.

- Severe renal insufficiency (GFR < 30ml/min/1.73 m2).

- EF less than 50%.

- Hepatic insufficiency GOT >2 times normal value.

- Uncontrolled blood pressure (>140/90 mmHg).

- Uncontrolled heart failure.

- Dissecting aortic aneurysm.

- Uncontrolled ventricular tachycardia or other dangerous ventricular arrhythmias (multifocal ventricular activity).

- Aortic or mitral valve disease.

- Recent systemic or pulmonary embolism.

- Active or recent thrombophlebitis.

- Acute infectious diseases.

- Uncontrolled supraventricular arrhythmias or tachycardias.

- Repeated or frequent ventricular ectopic activity.

- Moderate pulmonary hypertension.

- Ventricular aneurysm

- Uncontrolled diabetes, thyrotoxicosis, myxoedema

- Hepatic or renal insufficiency and other metabolic insufficiencies.

- Conduction disorders such as: Complete AV block. Left bundle branch block. Wolf-Parkinson-White syndrome.

- Fixed rate pacemaker.

- Severe anaemia.

- Psychoneurotic disorders.

- Neuromuscular, musculoskeletal and arthritic disorders that may limit activity.

## Data Analysis

The primary efficacy variable is METs-minutes/week. For the calculation of the power of the study, a 12% possible loss to follow-up was estimated. Thus, accepting an alpha risk of 0.05 in a bilateral test with 27 subjects in the control group and 27 patients in the intervention group, the power of the hypothesis test is 95% to detect as statistically significant the difference between the mean of 5000 in the first group and the mean of 4000 in the second group after rehabilitation. Based on the potential impact of the telerehabilitation intervention on compliance with the physical activity recommendation at 6-month follow-up and considering METs-minutes/week as the unit of measurement, it has been estimated that patients in the intervention group will achieve 5000 METs-minutes/week while in the conventional rehabilitation group they will achieve 4000 METs-minutes/week.

The characteristics of the patients in the two study groups will be described (applying the appropriate estimators according to the type of variables, such as means and confidence intervals, medians and interquartile ranges or proportions) and possible differences between the groups will be assessed using appropriate statistical tests of differences of means, distributions of ranks or proportions. The comparison of the primary variable and the secondary variables of the study in both groups at baseline, at four months and 10 months after the start of rehabilitation will be carried out using Pearson χ2 test for qualitative variables (Fisher exact test for dichotomous variable) or Student t-test for independent samples for quantitative variables (Mann-Whitney U test when parametric assumptions couldn’t be assumed). The treatment effects within groups (at four months or ten months) will be assessed using McNemar-Bowker test of symmetry for qualitative variables (McNemar test for dichotomous variable) or Student t-test for paired samples for quantitative variables (Wilcoxon signed-rank test when parametric assumptions couldn’t be assumed). The relationship between quantitative variables will be assessed by Pearson’s correlation coefficient (Spearman’s rank correlation coefficient when parametric assumptions couldn’t be assumed). Multiple linear regression models will be used to assess the significance and magnitude of the intervention effect controlling for possible differences in patient characteristics between groups. In general, backward-forward models will be used, initially constructing the complete models and excluding non-significant variables one at a time (p for variable removal: 0.10) but trying to include the previously removed variables after each exclusion (p for incorporation: 0.05. The analysis will be performed on an intention-to-treat basis. For the economic evaluation, the unit cost will be obtained by quantifying the consumption of resources and applying the official cost rates of the Valencian Health Agency or, if these are not available, they will be estimated by assessing the time and cost of the resources involved. Two-sided exact p-values will be calculated whenever possible and p-values ≤ 0.05 were considered statistically significant. Data will be analysed using IBM SPSS Statistics 22 and R 4.0.2 for Microsoft Windows.

## Calendar

Start of study: 01-10-2018

End of recruitment: 01-02-2019

End of study: 01-02-2020

Final report: 01-04-2020

Publication: 01-06-2020

## Financial Source

Catcronic Salut. S.L. (Nabelia)

Ronda Isaac Peral 9.

Parque Tecnológico de Valencia

46980 Paterna, Valencia.

# Index

[1. Summary 4](#_Toc76550906)

[1.1. Address and Protocol Identification 4](#_Toc76550907)

[1.2. Study Title 4](#_Toc76550908)

[1.3. Protocol Code 4](#_Toc76550909)

[1.4. Lead Researcher 4](#_Toc76550910)

[1.5. Centres where the study is planned to be carried out 4](#_Toc76550911)

[1.6. CEICs that evaluate it 4](#_Toc76550912)

[1.7. General Objective 4](#_Toc76550913)

[1.8. Secondary Objectives 5](#_Toc76550914)

[1.9. Design 5](#_Toc76550915)

[1.10. Disease or disorder under study 5](#_Toc76550916)

[1.11. Drug Data 5](#_Toc76550917)

[1.12. Population under study and total number of subjects 5](#_Toc76550918)

[1.13. Inclusion Criteria 6](#_Toc76550919)

[1.14. Exclusion Criteria 6](#_Toc76550920)

[1.15. Data Analysis 6](#_Toc76550921)

[1.16. Calendar 7](#_Toc76550922)

[1.17. Financial Source 7](#_Toc76550923)

[2. Index 8](#_Toc76550924)

[3. Study Justification 11](#_Toc76550925)

[4. Objectives 13](#_Toc76550926)

[4.1. General Objective 13](#_Toc76550927)

[4.2. Secondary Objectives 13](#_Toc76550928)

[5. Sources of Information and Scope 14](#_Toc76550929)

[6. Study Design 15](#_Toc76550930)

[6.1. Type of Study 15](#_Toc76550931)

[6.2. Population 15](#_Toc76550932)

[6.3. Definition of the study population: selection criteria 15](#_Toc76550933)

[6.4. Methods 16](#_Toc76550934)

[6.5. Data collection 22](#_Toc76550935)

[6.6. Outline of proceedings 24](#_Toc76550936)

[6.7. Treatment description 24](#_Toc76550937)

[7. Variables and measuring instruments 25](#_Toc76550938)

[7.1. Diagnostic criteria for the pathologies under study. 25](#_Toc76550939)

[7.2. Measuring instruments 25](#_Toc76550940)

[7.3. Variables 32](#_Toc76550941)

[8. Adverse Reactions 33](#_Toc76550942)

[8.1. Notification Period 33](#_Toc76550943)

[8.2. Adverse Reaction Definition 34](#_Toc76550944)

[8.3. Serious Adverse Reactions 34](#_Toc76550945)

[8.4. Register and Report of Adverse Events and Serious Adverse Events. 34](#_Toc76550946)

[9. Statistical Analysis 34](#_Toc76550947)

[9.1. Sample size calculation 34](#_Toc76550948)

[9.2. Description of the randomisation process 35](#_Toc76550949)

[9.3. Methodology of statistical analysis 35](#_Toc76550950)

[9.4. Economic evaluation: cost-effectiveness analysis 35](#_Toc76550951)

[10. Quality Control 36](#_Toc76550952)

[11. Monitoring 36](#_Toc76550953)

[12. Withdrawal of patients from study 37](#_Toc76550954)

[12.1. Criteria for definitive withdrawal from study 37](#_Toc76550955)

[12.2. Procedures and Consequences of a patient’s withdrawal from the study 37](#_Toc76550956)

[13. Ethical Aspects 38](#_Toc76550957)

[13.1. Accordance with the regulations in force 38](#_Toc76550958)

[13.2. Benefit-risk assessment for subjects participating in the study. 38](#_Toc76550959)

[13.3. Information and informed consent sheet. 39](#_Toc76550960)

[13.4. Data confidentiality 39](#_Toc76550961)

[13.5. Interference with individual doctor's prescribing habits 40](#_Toc76550962)

[13.6. Responsibility of all study participants. 40](#_Toc76550963)

[13.7. Clinical Research Ethics Committee (IRB) 41](#_Toc76550964)

[13.8. Study budget 41](#_Toc76550965)

[13.9. Insurance policy 42](#_Toc76550966)

[14. Final report and dissemination of results 42](#_Toc76550967)

[14.1. Monitoring and final reports 42](#_Toc76550968)

[14.2. Dissemination of results 42](#_Toc76550969)

[15. Bibliography 43](#_Toc76550970)

[16. ANNEXES 45](#_Toc76550971)

[ANEX 1. 46](#_Toc76550972)

[ANEX 2. 55](#_Toc76550973)

[ANEX 3. 56](#_Toc76550974)

[ANEX 4. 57](#_Toc76550975)

[ANEX 5. 58](#_Toc76550976)

# Rationale for the study

Cardiac rehabilitation after myocardial infarction or coronary syndrome, whether or not followed by percutaneous or surgical revascularisation, continues to be recognised in the latest guidelines and recommendations published by the major scientific societies as a class I^1,2^. These programs were proposed by the World Health Organisation in the 1960s to improve the quality of life and prognosis of patients with heart disease. The cardiac rehabilitation includes, in addition to a physical training program, guidelines for healthy nutrition, psychological counselling and stress management. This, together with pharmacological treatment, constitutes the best clinical practice in cardiovascular prevention for our patients. Despite this evidence, the number of patients joining these programs in Europe is still less than 30%, a figure that is reduced in Spain to a symbolic 3%, according to data from the EUROASPIRE III study^3^. According to this study, 17% of those surveyed after a coronary event still smoked; 35% were obese; 56% had hypertension and 51% had dyslipidaemia. Considering that the measures carried out in these rehabilitation programs are very cost-effective in reducing cardiovascular risk, it is difficult to understand why they have not received greater impetus from health authorities and professionals involved so far. Instead, the impact of pharmacological prevention measures, enhanced in recent years, has contributed to drastically improve the prognosis of patients after acute coronary syndrome, partly diminishing the weight of evidence for the benefit of cardiac RHB programs^4,5^. In two of the latest prospective trials, cardiac BHR programs failed to demonstrate positive results on the primary endpoint (total mortality, myocardial infarction and hospital readmission), although they were superior on some secondary endpoints such as non-fatal infarction or length of hospital stay^6,7^. Taylor et al, in 2004 noted that the benefit of cardiac RHB with exercise alone was 16% in terms of cardiovascular mortality. If it was also incorporated into a comprehensive program, including risk factor control and educational measures, the benefit amounted to 24% ^8^. In a later published meta-analysis, the authors estimated that half of the 28% reduction in cardiac mortality achieved was attributable to the reduction in major risk factors and, therefore, there was no independent risk reduction due to physical exercise alone ^9^. Martin et al, in a prospective cohort study, showed that subjects (n=2900) who completed a full rehabilitation program had a lower risk of death, cardiac and overall hospitalisation compared to those subjects (n=554) who did not^10^. Suaya et al, in an analysis to control for a number of variables in participants and non-participants in BHR programs, among 60,000 coronary subjects over 65 years of age included in MEDICARE, observed a 21-34% reduction in 5-year mortality in BHR program users compared to non-users^11^. Unfortunately, in this multidisciplinary program the impact of physical exercise on mortality reduction was not specifically analysed. In any case, it seems that the benefit of physical exercise is inseparable from the other measures. Therefore, the European and American guidelines on cardiovascular prevention describe the recommendations for physical activity and participation in rehabilitation programs in separate sections. Moreover, the benefit of cardiac rehabilitation programs is directly related to the length of time the patient remains in the program. This relationship has been analysed in 3,161 patients with a recent coronary event included in the Medicare database, who attended at least one rehabilitation session. After 4 years of follow-up, patients who attended 36 sessions had a 14% lower risk of death and a 12% lower risk of myocardial infarction than those who attended 24 sessions; a 22% lower risk of death and a 23% lower risk of myocardial infarction than those who attended 12 sessions; and 47% lower risk of death and 31% lower risk of myocardial infarction than those who attended only one session. However, only 18% of patients completed the maximum of 36 sessions^12^. On the other hand, it appears that health-related quality of life does not differ in relation to the duration of the 6-month rehabilitation program compared to a 12-month rehabilitation program^13^. Current rehabilitation programs based on inpatient supervision have several limitations. One of the most important is the lack of adherence in the medium and long term. At the end of phase II, few patients adopt the advice given for the rest of their lives and continue to do physical activity at the required intensity (phase III). There is a direct relationship between the degree of physical training maintained and cardiovascular morbidity and mortality^14,15^ and therefore some of the benefits achieved are reversed over time. To overcome these limitations, modifications and new strategies to existing programs are probably required. Outpatient rehabilitation programs are being evaluated in recent years as alternative strategies with good results^16^. The development of telematic applications and Internet-based platforms constitute the fundamental tools for carrying out these home-based rehabilitation programs^17^. There are some experiences in which their feasibility has been demonstrated, increasing adherence to physical activity^18^. Their use in conditions of distance from the hospital and their equal or lower cost in some cases are other possible advantages^19^ . The present study is aimed at validating a new Integrated Telerehabilitation System as support for post-infarction rehabilitation, optimising it and testing its usefulness in terms of improving adherence to physical exercise and cardiovascular risk parameters. It should also be analysed whether there are limitations to the use of these technologies by older people with no previous experience in the use of new communication technologies. The economic analysis will make it possible to estimate whether there are added advantages such as cost containment. The importance of these innovations has led the European Union to allocate funds within the Seventh Framework Program to promote new technological developments. The platform and application used in this trial, with the appropriate modifications, are already being used in the follow-up of heart transplant patients ^20^. In a context of rising health system costs, it is important to raise patient awareness of the responsibility of maintaining health by adopting healthier lifestyle habits. Moreover, these innovations in rehabilitation programs can encourage motivation and act synergistically, together with other pharmacological measures, to achieve the prevention objectives that require so much work and effort on the part of all the actors involved.

# Objectives

## General Objective

Improvement in compliance with physical activity at 10 months follow-up, taking METs-minutes/week as the unit of measurement.

## Secondary Objectives

Verify the improvement of the following parameters after the intervention:

A. Stress test:

● Demonstrate increase in VO2max.

● Demonstrate increase in exertion time.

● Demonstrate increase in workload.

● Decrease the presence of symptoms.

B. Analytical:

● Obtain improvement in lipid profile.

● Decrease in CRPus.

C. Lifestyle:

● Demonstrate increased adherence to Mediterranean diet.

● Demonstrate improved outcomes in psychological state.

● Demonstrate increased willingness to quit smoking and smoking cessation.

● Demonstrate a decrease in the sedentary lifestyle indicator.

● Demonstrate an earlier return to work.

D. Arterial stiffness

Improving carotid-femoral pulse wave velocity measurement

E. Socio-economic variables

To evaluate the cost per patient/month in the control cases of conventional rehabilitation and extended tele-rehabilitation intervention.

# Sources of Information and Scope

The information sources for the present study are:

● Participating investigators.

● Data from the patient's medical history.

● Quality of life questionnaires (EQ-5D), Adherence to Mediterranean diet, physical activity (IPAQ), anxiety-depression questionnaire (HAD).

● Analytical data.

● Pulse wave velocity.

● Data derived from stress tests (treadmill).

● Socio-occupational data

The two rehabilitation protocols that are compared are those that have been adopted in each hospital centre. At La Fe hospital it has been in operation for years and at the Arnau de Vilanova hospital it is to be initiated with the present study. There are no additional tests except for the completion of the questionnaires mentioned above.

All necessary data will be collected in a data collection logbook (DCL).

Patients will be recruited from the Cardiology ward, in both hospitals.

# Study Design

## Type of Study

Phase III clinical trial, feasibility, controlled and randomised, with two branches (intervention group by cardiac telerehabilitation and conventional rehabilitation control group in the hospital), developed in the cardiology service of the Hospital Arnau de Vilanova and Hospital Universitari i Politècnic La Fe.

The trial will be randomised, as the inclusion of each patient will be randomised by a unit external to the researchers, which will establish to which group the patient will be assigned. However, the characteristics of the interventions do not allow the study to be blinded either for the patient or for the professional. However, the analyses, stress tests and shuttle tests will be performed in a masked manner without the assigned group being identifiable by the investigators carrying out the examinations.

Sixty patients after acute coronary syndrome from two hospitals will be included.

## Population

Men and women, with similar demographic characteristics, who have suffered an acute coronary event (unstable angina, NSTEACS or STEACS), regardless of whether they have undergone revascularisation or not, and who do not have angina or residual electrical ischaemia on a stress test 7-10 days after hospital discharge, will be included. The 60 patients are divided into two groups:

1. Control group A comprising 30 patients from Hospital Universitario Politécnico La Fe and Hospital Arnau de Vilanova in Valencia, who will undergo conventional in-patient rehabilitation for 8 weeks at Hospital La Fe.
2. Intervention group B comprising 30 patients from the Hospital Arnau de Vilanova and Hospital Universitario y Politécnico La Fe de Valencia, who will carry out a telerehabilitation program with the support of the Integral Telerehabilitation System (ITS) at the Hospital Arnau de Vilanova.

## Definition of the study population: selection criteria

1. Inclusion Criteria

- Patients after uncomplicated acute coronary syndrome of both sexes.
- Completion of a maximal or symptom-limited exercise test without angina or electrical ischaemia.
- Age equal to or less than 72 years.

1. Exclusion Criteria

- Refusal of informed consent
- At the investigator's judgement, primarily due to advanced biological age.
- Severe renal insufficiency (GFR < 30ml/min/1.73 m2).
- EF less than 50%.
- Hepatic insufficiency (GOT >2 times normal value).
- Uncontrolled blood pressure (>140/90 mmHg).
- Uncontrolled heart failure.
- Dissecting aortic aneurysm.
- ventricular tachycardia or other dangerous ventricular arrhythmias that are not controlled (multifocal ventricular activity).
- Aortic or mitral valve disease.
- Recent systemic or pulmonary embolism.
- Active or recent thrombophlebitis.
- Acute infectious diseases.
- Uncontrolled supraventricular arrhythmias or tachycardias.
- Repeated or frequent ventricular ectopic activity.
- Moderate pulmonary hypertension.
- Ventricular aneurysm.
- Uncontrolled diabetes, thyrotoxicosis, myxoedema,
- Hepatic or renal failure and other metabolic insufficiencies.
- Conduction disorders such as: Complete AV block. Left bundle branch block. Wolf-Parkinson-White syndrome.
- Fixed rate pacing.
- Severe anaemia.
- Psychoneurotic disorders.
- Neuromuscular, musculoskeletal and arthritic disorders that may limit activity.

1. Withdrawal Criteria

- Withdrawal of consent to continue the study.
- At the investigator's discretion if appropriate for the patient.
- Need to modify lipid-lowering treatment.
- Unwillingness to continue the study or loss due to dropout.
- Clinical events.
- Other reasons at the discretion of the investigator.

## Methods

After explaining the fundamentals of the study to the patients and accepting their participation, they will sign the informed consent form. The first procedure will be to perform a symptom-limited treadmill exercise test with oxygen consumption, seven to ten days after hospital discharge, using the Bruce protocol. If the test results allow inclusion in the study, demographic data, clinical history, physical examination and analytical data will be completed in the data collection notebook. The baseline surveys will be completed, the procedures to be followed will be explained, as well as the schedule of the various activities included in each of the groups. Patients shall receive medication as deemed appropriate by the physician at the time of discharge from hospital, with the initial goal of achieving LDL of 70 mg/dl or less, blood pressure of 140/90 mmHg or less and, where appropriate, glycosylated haemoglobin of less than 7%.

The follow-up period will be 10 months in both groups. The differences in follow-up are detailed below.

At the end of the 10-month follow-up period, all subjects included in the study will undergo a stress test, final analysis and completion of the various outcome evaluation surveys.

Control Group A.

Patients in Control Group A will come to the hospital twice a week during the 8 weeks of the conventional rehabilitation program. Eight sessions will be devoted to physical exercise and another eight sessions to education in various aspects. At the end of the hospital phase, outpatient follow-up by primary and specialised care will be carried out. Subjects will visit the hospital a total of 16 times during 8 weeks to perform the exercises included in the hospital protocol, supervised by a physiotherapist and a nurse from the Cardiology Service.

The protocol includes:

Preparation to start the activity (Warm-up).

15 minutes of low impact cardiovascular exercise plus stretching and flexibility.

Stretching:

8-10 seconds interspersed with activities that maintain heart rate such as walking in place.

Musculature involved: triceps sural, hamstrings, quadriceps, deltoids, paravertebral and pectoralis.

The circuit:

The circuit is designed to cater for all patients with varying functional abilities. It provides the opportunity to increase cardiovascular work time. It is organised as explained in the following diagram:

| **LEVEL** | Group session content |
| --- | --- |
| 1 | 1 CV station followed by 1 min. Of AR |
| 2 | 2 CV stations followed by 1 min. Of AR |
| 3 | 3 CV stations followed by 1 min. Of AR |
| 4 | 4 CV stations followed by 1 min. Of AR |
| 5 | 5 CV stations followed by 1 min. Of AR |

- Warm-up
- Together with physiotherapist
- CV work (circuit)
- Individual work (levels)

**Part 1**

**15 min**

**Part 2**

**30 min**

- Cooldown + Stretching
- With Physiotherapist

**Part 3**

**15 min**


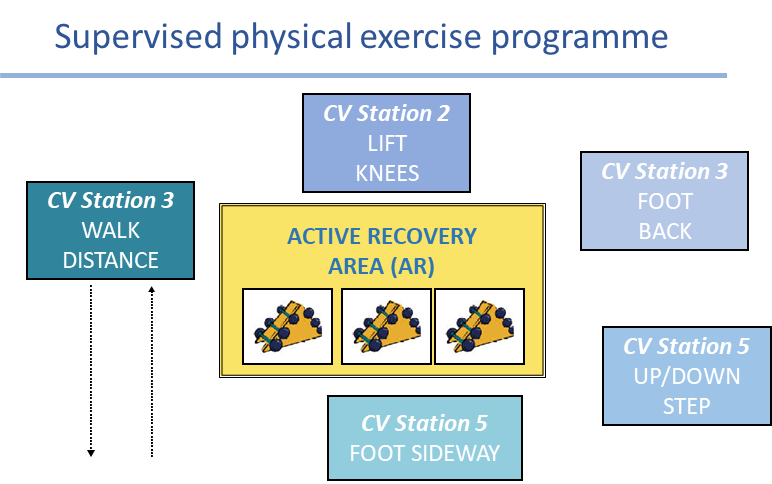


Educational program consisting in 8 talks about the following topics:

Supervised physical exercise program


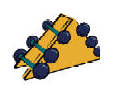

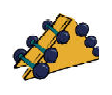

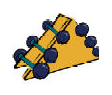


**ACTIVE RECOVERY AREA (AR)**

***CV Station 2***

LIFT

KNEES

***CV Station 3***

FOOT

BACK

***CV Station 5***

UP/DOWN

STEP

***CV Station 5***

FOOT SIDEWAY

***CV Station 3***

WALK

DISTANCE

Supervised physical exercise programme


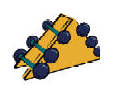

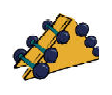

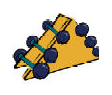


**ACTIVE RECOVERY AREA (AR)**

***CV Station 2***

LIFT

KNEES

***CV Station 3***

FOOT

BACK

***CV Station 5***

UP/DOWN

STEP

***CV Station 5***

FOOT SIDEWAY

***CV Station 3***

WALK

DISTANCE

- Hypertension and cholesterol

- Coronary heart disease

- Cardiovascular drugs

- Heart disease and sex

- Diabetes

- Physical exercise

- Diet

- Tabaco

Intervention Group B.

The patients in Intervention Group B, included in the telerehabilitation program, will attend the hospital twice a week for 2 consecutive weeks, carrying out three sessions of physical exercise and four sessions of education on the same subjects as control group A. Subsequently, they will follow the guidelines programed in the ITS by carrying out their guided physical activities and adherence to the guidelines for management of their risk factors until the end of the study period. All the data generated are recorded in the professional application.

The program consists of two phases: an outpatient phase, in which the patient will attend the hospital 4 times during two weeks; and a second long-term phase of 10 months, where the patient will carry out the entire rehabilitation and prevention program with total independence.

Hospital phase: Starting on the first day, each individual in the patient team is provided with the ITS system described in the materials section. The patient is instructed to perform the programed physical activity and how to use the instrumentation, consisting of the application on the smartphone and the web connection to access the educational content and control of recommended hygienic and dietary measures, as well as monitoring of the pharmacological treatment. Ambulatory physical activity consists of walking at a sustained pace, until the effective heart rate equal to 75% of the maximum heart rate, obtained in the baseline stress test, is reached. The intensity of the exercise will be individually adapted according to the result of this same test. The hospital stay will be about two and a half hours, according to the following schedule:

1st session (Monday)

- Data collection: 60m

- Lecture: Explanation of the program and how the system works: 30m

- First exercise session: 60m

2nd session (Thursday)

- Clarification of doubts about how the system works: 30m

- Talk: Coronary heart disease and cardiovascular risk factors: 40m

- Second exercise session: 60m

3rd session (alternate weeks, Mondays) (12 patients)

- Talk: Anxiety and depression. Strategies to resolve conflicts: 45m.

- Talk: Sexuality and smoking: 45m

- Sketches humour: 30m

4th session (Thursdays)

- Talk: Hypertension and diabetes: 30m

- Talk: Diet and nutrition: 30m

- Third exercise session: 60m

Ambulatory phase: from week 3 until the end of the trial at week 40, the patient should perform his physical exercise routines 4 times per week monitored by the ITS. These routines can be performed by walking outdoors. The patient's application on the mobile phone, allows the heart rate to be calculated for each exercise session. The system alerts about possible deviations from the programed training heart rate so that the patient can adjust their physical activity to achieve the recommended frequency. All this data is also transmitted to the platform for subsequent analysis by the professional. The application is not only a means of controlling and monitoring the quality of physical exercise, but also fundamentally a tool to assist in cardiovascular prevention in a comprehensive way. This means that the patient's cardiovascular risk factors will be recorded, in order to help the patient modify their lifestyle and correct or minimise the risk factors that contribute to disease progression. The support website includes personalised aids (information, education, motivational messages) so that the patient can self-manage their daily life and feel protected and in control.

The educational program will consist of 6 lectures on the following topics:

- Telerehabilitation program

- Information on coronary heart disease and cardiovascular risk factors.

- Anxiety and depression in the context of an acute coronary event.

- Sexuality and return to work after an acute coronary event

- Heart-healthy diet and the benefits of physical exercise.

- Smoking.

The technology available to patients in the telerehabilitation group includes the following:

A. Comprehensive telerehabilitation system

The Integral Telerehabilitation System (ITS) is a software and services platform created by Nabelia (http://nabelia.com/es/) to establish Cardiac Prevention and Rehabilitation Programs (CPRP) aimed at helping the management and personalised management of cardiovascular risk factors, with the highest quality but in a cost-effective manner for the healthcare centre and professionals. Its objective is to provide continuous, long-term care and support to patients with CVD during their recovery process.

The ITS to be configured for the study consists of the following elements:

1) Web-based application for the practitioner in the hospital, which allows to:

o Advise the patient on their personal care plan

o Create/modify standard plans to suit individual needs and personal goals

o Establish the patient's risk profile and targets for improvement

o Assess overall and CVRF status by means of aggregate indicators

o Long-term monitoring of the evolution of CVRFs and events that have occurred

o Advise the patient on long-term CVRF self-management strategies

For its part, the system is able to:

o Dynamically stratify patients according to risk, and supports the practitioner in assessing the need for unscheduled interventions, generating alerts when the parameters entered are outside the pre-set range.

o Provides decision support for the hospital or risk management service provider.

o Provides a workflow of procedures and follow-up interventions, and guides and reminds the patient of each step to improve adherence.

o Automatically generates advice and motivational messages to the patient based on the risk factors and circumstances present at any given time.

2) Patient applications

*Mobile phone software with the following functions:*

o Tasks

- Scheduled exercise sessions

- Medication reminders

- Reminder to take measurements (weight, blood pressure, heart rate, etc.)

o My status: personal information of:

- Care plan: medication, measurements, and physical activity

- Status of the risk factors present, objectives, evolution, and advice

- Results of the last data added (measurements, questionnaires, tasks carried out).

o Messages: Inbox of messages from the system and messages generated by professionals in a personalised way for a specific patient.

o Training monitor: guides the patient in carrying out their exercise.

*Patient website with the following services:*

o Profile and personal data.

o Evolution of aggregate indicators. History of the measurements introduced, and tasks carried out. Achievements.

o Access to health information aimed at certified patients: Salupedia, news, blogs, youtube, etc.

o Health literacy programs.

o Motivational programs.

3) Patient sensor equipment

The cardiac monitor is a comfortable compression garment that is designed for easy donning and adaptable to various physical conformations of the patient's chest. The garment has two textile electrodes from which a monopolar ECG signal is recorded. The electronics built into the garment processes this signal and transmits the data - using Bluetooth connection - to the mobile phone application. The measured data are:

o Instantaneous and average heart rate.

o Calories. METs, distance, speed, GPS position

o Exercise quality index (% of target achievement).

## Data collection

The following data shall be collected on all patients at baseline, at 4 months and at 10 months.

1. Demographic data: initials of name, age, sex, identification number).

2. Clinical data: STEMI, NSTEMI, unstable angina, stable angina, number of vessels, number of stents, complete revascularisation, active smoking, ex-smoker, hypertension, dyslipidaemia, diabetes mellitus, left ventricular ejection fraction, beta-blockers, ACEI/ARBs, statins, dual antiplatelet therapy.

3. Socio-occupational data: active worker, return to work after discharge.

1. Physical examination: systolic blood pressure, diastolic blood pressure, heart rate, body mass index (BMI), waist circumference, hip circumference.
2. Analytical data: glucose, creatinine, total cholesterol, LDL cholesterol, HDL cholesterol, triglycerides, CRPus, HbA1c.
3. Pulse wave velocity.
4. Surveys: EQ-5D, IPAQ, HAD, Adherence to Mediterranean diet, Fagerström test (if active smoking).
5. Clinical events: emergency department, readmission, revascularisation.

The following data will only be collected at baseline and at 10 months.

1. Exercise test: VO2max, exercise time, maximum heart rate, maximum blood pressure, recovery time, ST decline, angor.

The following data will only be collected from patients in the intervention group:

1. Every 15 days: weight, smoking, waist circumference, systolic pressure, diastolic pressure, heart rate, visual anxiety-depression thermometer, fruit consumption, vegetables, wine/beer consumption.

2. At 4 and 10 months: survey of satisfaction with the application.

## Outline of proceedings

|  | **Start** | **4 months** | **10 months** |
| --- | --- | --- | --- |
| **INFORMED CONSENT** | X |  |  |
| **FILIATION DATA** | X | X | **X** |
| **CLINICAL DATA** | X |  |  |
| **SOCIO-OCCUPATIONAL DATA** | X | X | X |
| **PHYSICAL EXPLORATION** | X |  |  |
| **PWV** | X |  | X |
| **BLOOD TEST** | X | X | X |
| **STRESS TEST** | X |  | X |
| **PHYSICAL WORKING CAPACITY** | X |  | X |
| **SURVEY** | X |  | X |
| **CLINICAL EVENTS** | X | X | X |
| **COST-EFFECTIVENESS ANALYSIS** |  |  | X |

## Treatment description

Not applicable. No drug is evaluated in either of the two groups.

# Variables and measuring instruments

## Diagnostic criteria for the pathologies under study.

Unstable angina. This is a clinical syndrome that presents with chest pain in response to myocardial ischaemia, associated or not with ST-segment depression or elevation or T-wave inversion, transiently, without elevation of troponin or CK-MB.

NSTEACS. Clinical syndrome of ischaemic chest pain together with ST-segment depression or elevation or T-wave inversion, transient but somewhat more persistent, with troponin or CK-MB elevation.

STEACS. A clinical syndrome of chest pain secondary to myocardial ischaemia associated with persistent ST-segment elevation and elevated troponin or CK-MB.

## Measuring instruments

**A. Analytical data**

Glucose, creatinine, total cholesterol, LDL cholesterol, HDL cholesterol, triglycerides, CRPus, HbA1c

**B. Survey**

**a. Questionnaire on adherence to the Mediterranean diet:**

| **1** Do you use olive oil as your main cooking fat? | Yes | 1 point |
| --- | --- | --- |
| **2** How much olive oil do you consume in total per day (including frying, eating out, salads, etc.)? | 2 or more tablespoons | 1 point |
| **3** How many servings of vegetables do you eat per day (1 serving = 200g. Garnishes or side dishes are equivalent to  ½ portion)? | 2 or more (at least 1 of them in raw salad) | 1 point |
| **4** How many pieces of fruit (including fruit juice) do you eat per day? | 3 or more | 1 point |
| **5** How many portions of red meat, hamburgers, sausages or cold meats do you eat per day (1 portion = 100-150 g)? | Less than 1 | 1 point |
| **6** How many servings of butter, margarine or cream do you consume per day (single portion = 12 g)? | Less than 1 | 1 point |
| **7** How many carbonated and/or sweetened beverages (soft drinks, colas, tonic, bitters) do you consume per day? | Less than 1 | 1 point |
| **8** Do you drink wine and how much every week? | 3 or more glasses | 1 point |
| **9** How many portions of pulses do you consume per week (1 plate or  portion = 150 g)? | 3 or more | 1 point |
| **10** How many portions of fish/seafood do you consume per week (1 plate, piece or portion = 100-150 g fish or 4-5 pieces or 200 g seafood)? | 3 or more | 1 point |
| **11** How many times do you eat commercial confectionery (not homemade,  such as biscuits, custards, sweets, pastries, cakes) per week? | Less than 3 | 1 point |
| **12** How many times do you eat nuts and dried fruit per week (1 portion = 30 g)? | 1 or more | 1 point |
| **13** Do you prefer chicken, turkey or rabbit meat to beef, pork, hamburger or sausage (chicken, turkey or rabbit: 1 piece or portion 100-150 g)? | Yes | 1 point |
| **14** How many times a week do you eat cooked vegetables, pasta, rice or other dishes seasoned with tomato, garlic, onion or leek sauce simmered with olive oil (sofrito)? | 2 or more | 1 point |

Assessment:

a. If the result is equal to or less than 8 points >>>> it is in your interest to improve.

b. If the result is between 9-12 points >>> you are doing well although you can still improve

c. If the result is 13 or 14 points >>> continue in the same direction.

**b. HAD Questionnaire. Hospital, anxiety, depression**

This test is intended to determine how you have been feeling in the last week, although the questions are in the present tense. You have to choose between one of four possibilities regarding the question asked, circling the chosen answer. (A = Anxiety) (D = Depression)

1. I am tense or nervous (A)

(0) Never A

(1) Sometimes

(2) Many times

(3) Everyday

1. I still enjoy what I once loved (D)

(0) Never D

(1) Sometimes

(2) Many times

(3) Everyday

1. I have a feeling of fear, as if something horrible was about to happen (A)

(0) Never A

(1) Sometimes

(2) Many times

(3) Everyday

1. I can laugh and see the bright side of things (D)

(0) Never D

(1) Sometimes

(2) Many times

(3) Everyday

1. I am full of worries (A)

(0) Never A

(1) Sometimes

(2) Many times

(3) Everyday

1. I feel happy (D)

(0) Never D

(1) Sometimes

(2) Many times

(3) Everyday

1. I can sit quietly and feel relaxed (A)

(0) Never A

(1) Sometimes

(2) Many times

(3) Everyday

Assessment:

1. If the result is between 0 and 7 it does not indicate a case of either anxiety or depression.

b. If the result is between 8 and 10 it would be a doubtful case.

c. If the result is above 11 it is probably a case in each of the subscales.

**c. Physical Activity Questionnaire (IPAQ)**

1. During the last 7 days, on how many days do I do intense physical activities such as heavy lifting, digging, aerobic exercise or fast cycling?

Days per week (please indicate the number)

No heavy physical activity (skip to question 3)

2. How much time in total did you usually spend doing vigorous physical activity on any one of those days?

Please indicate how many hours per day

Please indicate how many minutes per day

Don't know/not sure

3. During the last 7 days, on how many days did you do moderate physical activity such as carrying light weights, or cycling at a regular speed? Do not include walking

Days per week (indicate number)

No moderate physical activity (skip to question 5)

4. How much time did you usually spend in total in moderate physical activity on one of those days?

Indicate how many hours per day

Indicate how many minutes per day

Don't know/not sure

5. During the last 7 days, on how many days did you walk for at least 10 minutes at a time?

Days per week (give number)

No walking at all (skip to question 7)

6. How much time in total did you usually spend walking on any one of those days?

Indicate how many hours per day

Indicate how many minutes per day

Don't know/not sure

7. During the last 7 days, how much time did you spend sitting during a working day?

Please indicate how many hours per day

Please indicate how many minutes per day

Don't know/not sure

**EVALUATION:**

1. Walking: 3'3 METs x minutes of walking x days per week (e.g. 3'3 x 30 minutes x 5 days = 495 METs).

2. Moderate Physical Activity: 4 METs X minutes x days per week

3. Vigorous Physical Activity: 8 METs X minutes x days per week

Then add up the three values obtained:

**Total = walking + moderate physical activity + vigorous physical activity**

**CLASSIFICATION CRITERIA:**

Moderate Physical Activity:

1. 3 or more days of vigorous physical activity at least 20 minutes per day.

2. 5 or more days of moderate physical activity and/or walking at least 30 minutes per day.

3. 5 or more days of any combination of walking, moderate or vigorous physical activity achieving at least a total of 600 METs*.

Vigorous Physical Activity:

1. Vigorous Physical Activity at least 3 days per week achieving a total of at least 1500 METs*.

2. 7 days of any combination of walking, moderate physical activity and/or vigorous physical activity, achieving a total of at least 3000 METs*.

** Unit of measurement of the test.*

RESULT: ACTIVITY LEVEL (tick as appropriate)

HIGH LEVEL

MODERATE LEVEL

LOW OR INACTIVE LEVEL

**d. EQ-5D Questionnaire**

Please check the statement in each section that best describes your health status today.

**Mobility**

I have no problems walking

I have some problems walking

I have to stay in bed

**Personal Care**

I have no problems with personal care

I have some problems to wash and dress myself

I am uncapable of washing and dressing myself

**Everyday Activities** (e.g., working, studying, chores, family activities)

I have no problem performing my everyday activities

I have some problems performing my everyday activities

I am uncapable of performing my everyday activities

**Pain/Discomfort**

I have no pain nor discomfort

I have moderate pain or discomfort

I have a lot of pain or discomfort

**Anxiety/Depression**

I am not anxious or depressed

I am moderately anxious or depressed

I am very anxious or depressed

To help people describe how good or bad their state of health is, we have drawn a thermometer-like scale on which the best imaginable state of health is marked 100 and the worst imaginable state of health is marked 0.

Please draw a line from the box that says "your health today" to the point on the scale that, in your opinion, indicates how good or bad your health is today.


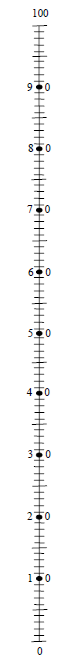


**Your health status today**

Best possible health

Worst possible health

**C. Pulse Wave Velocity (PWV)**

Carotid-femoral PWV is determined by ultrasound using an iE33 (Philips Medical Systems, USA), with an S 113 transducer (7-10 MHz). First, the time between the R wave of the ECG and the onset of the systolic Doppler wave in the right common carotid artery is measured. Subsequently, the time between the ECG R-wave and the foot of the systolic Doppler wave in the radial and femoral artery is measured. For each measurement, 6 cardiac cycles are averaged. The distance between measurement points, marked on the skin, is measured with a specially designed compass avoiding variability attributed to body contour. The PWV is calculated as the distance between the measurement points divided by the carotid-femoral or carotid-radial time difference (transit time of the pulse wave between the two points), in metres per second. Subjects remain at rest in a quiet, temperature-controlled room 10 minutes prior to measurements, which will be performed in the morning and according to published procedures 6. All measurements will be performed by the same scout.

## Variables

1. Principal Variable

Since the main objective is adherence to a physical activity program, the main variable of the study is the METS-minute/week measure. Obtained from the IPAQ questionnaire.

1. Secondary variables

1. Clinical data: age, sex, STEMI, NSTEMI, unstable angina, stable angina, number of vessels, number of stents, complete revascularisation, active smoking, former smoker, hypertension, dyslipidaemia, diabetes mellitus, left ventricular ejection fraction, beta-blockers, ACE inhibitors/ARBs, statins, dual antiplatelet therapy.

2. Socio-occupational data: active worker, days until return to work after discharge.

3. Physical examination: systolic blood pressure, diastolic blood pressure, heart rate, body mass index (BMI), waist circumference, hip circumference.

4. Exercise test: VO2max, exercise time, maximal heart rate, maximal blood pressure, recovery time, ST decline, angor.

5. Pulse wave velocity.

6. Analytical data: glucose, creatinine, total cholesterol, LDL cholesterol, HDL cholesterol, triglycerides, CRPus, HbA1c.

# Adverse Reactions

All adverse reactions or suspected adverse reactions should be recorded on the Data Collection Notebook sheets in the Adverse Reactions section.

For all adverse reactions, the investigator should seek and obtain sufficient information to determine the outcome of the adverse reaction and to assess whether it meets the criteria for classification as a serious adverse reaction (see section on "Serious Adverse Reactions") which should be reported immediately to the competent authorities. The investigator should obtain sufficient information to determine the causality of the adverse reaction.

## Notification Period

Serious adverse events will be reported by the principal investigator within 1 day to the study monitor or Nabelia staff by telephone or FAX. Incidents and consequences of the serious adverse event should be reported in writing within 4 days. The adverse reaction form should be completed for fatal cases and, whenever possible, all deaths occurring within 30 days after the end of the study should be reported.

According to current legislation, the study sponsor must report serious adverse events to the Spanish Agency for Medicines and Health Products (AEM) and to the Ethics Committee of the corresponding hospital.

The maximum notification period shall be 15 calendar days from the time of knowledge of the suspected adverse reaction. When the suspected serious and unexpected adverse reaction has resulted in the death of the subject, or has endangered his life, the sponsor shall inform the AEMPS within a maximum of 7 calendar days from the moment the sponsor becomes aware of the case. This information should be completed, if possible, within the following 8 days. For adverse reactions occurring in clinical trials conducted in Spain, the number assigned by the EMA to the corresponding protocol must be explicitly mentioned.

The sponsor shall notify the CEIC and the competent bodies of the Autonomous Community individually and within a maximum of 15 days of all suspected adverse reactions which are both serious and unexpected associated with the investigational product. This maximum period shall be 7 days in the case of suspected life-threatening or life-threatening adverse reactions.

The safety report shall either be a part of the corresponding annual and final report or be prepared independently. In either case, the annual and final reports shall contain in tabular form all adverse events detected in the trial up to the time of their preparation.

For all aspects related to adverse events, the guidelines established by the AEMPS shall be followed.

## Adverse Reaction Definition

An adverse reaction is any undesirable event that occurs to a patient who has started the rehabilitation program.

Examples of adverse reactions include the following:

- Clinically significant symptoms and signs.

- Changes in physical examination findings.

## Serious Adverse Reactions

A serious adverse reaction is any unwanted medical event that:

- Causes death;

- Is life-threatening (immediate risk of death);

- results in hospitalisation or prolonged hospitalisation;

- Causes persistent or significant disability or incapacity;

- Is considered medically important by the investigator or sponsor.

## Register and Report of Adverse Events and Serious Adverse Events.

Adverse events shall be recorded in the appropriate section of the data collection logbook (DCL).

# Statistical Analysis

## Sample size calculation

## The sample size is determined by the number of patients eligible for inclusion in the study who are seen in each hospital during the inclusion period. Thirty patients will be included in each of the following groups: control group, conventional rehabilitation group and telerehabilitation group. For the calculation of the power of the study, a 12% possible loss to follow-up has been estimated. Thus, accepting an alpha risk of 0.05 in a bilateral test with 27 subjects in the control group and 27 patients in the intervention group, the power of the hypothesis test is 95% to detect as statistically significant the difference between the mean of 5000 in the first group and the mean of 4000 in the second group after rehabilitation. Based on the potential impact of the telerehabilitation intervention on compliance with the physical activity recommendation at 10 months follow-up, and considering METs-minutes/week as the unit of measurement, it has been estimated that patients in the intervention group will perform 5000 METs-minutes/week while in the conventional rehabilitation group they will perform 4000 METs-minutes/week. It is estimated that physical activity in the non-rehabilitation group will be significantly lower.

## Description of the randomisation process

After agreeing to participate in the study, subjects will be assigned to follow the conventional rehabilitation or telerehabilitation program, according to a block randomisation, estimated using a conventional statistical program.

Subject eligibility will be established prior to allocation to rehabilitation type and inclusion will be sequential. If a subject withdraws from the study his/her randomisation number will not be re-used and re-entry into the study will not be allowed.

## Methodology of statistical analysis

The characteristics of the patients in the two study groups will be described (applying the appropriate estimators according to the type of variables, such as means and confidence intervals, medians and interquartile ranges or proportions) and possible differences between the groups will be assessed using appropriate statistical tests of differences of means, distributions of ranks or proportions. The comparison of the primary variable and the secondary variables of the study in both groups at baseline, at four months and 10 months after the start of rehabilitation will be carried out using Pearson χ2 test for qualitative variables (Fisher exact test for dichotomous variable) or Student t-test for independent samples for quantitative variables (Mann-Whitney U test when parametric assumptions couldn’t be assumed). The treatment effects within groups (at four months or ten months) will be assessed using McNemar-Bowker test of symmetry for qualitative variables (McNemar test for dichotomous variable) or Student t-test for paired samples for quantitative variables (Wilcoxon signed-rank test when parametric assumptions couldn’t be assumed). The relationship between quantitative variables will be assessed by Pearson’s correlation coefficient (Spearman’s rank correlation coefficient when parametric assumptions couldn’t be assumed). Multiple linear regression models will be used to assess the significance and magnitude of the intervention effect controlling for possible differences in patient characteristics between groups. In general, backward-forward models will be used, initially constructing the complete models and excluding non-significant variables one at a time (p for variable removal: 0.10) but trying to include the previously removed variables after each exclusion (p for incorporation: 0.05. The analysis will be performed on an intention-to-treat basis. For the economic evaluation, the unit cost will be obtained by quantifying the consumption of resources and applying the official cost rates of the Valencian Health Agency or, if these are not available, they will be estimated by assessing the time and cost of the resources involved. Two-sided exact p-values will be calculated whenever possible and p-values ≤ 0.05 were considered statistically significant. Data will be analysed using IBM SPSS Statistics 22 and R 4.0.2 for Microsoft Windows.

## Economic evaluation: cost-effectiveness analysis

The study includes a cost-effectiveness analysis to assess the costs and impact on health-related quality of life of the telerehabilitation intervention compared to in-hospital rehabilitation and the control group. The cost analysis will be carried out from the funder's perspective, including direct health care costs (pathology-related emergency visits, hospitalisations for the same cause, diagnostic and therapeutic tests such as revascularisations...) during follow-up. It will also include the costs derived from the intervention, which in the case of conventional rehabilitation, the programd schedule includes 16 rehabilitation visits, 8 rehabilitation sessions (with a physiotherapist, a nurse and a nursing assistant per session) and 8 educational talks. In the telerehabilitation group, the schedule includes 4 rehabilitation visits, 3 sessions (with a physiotherapist and a nurse) and 4 educational sessions. Unscheduled visits and sessions during the follow-up will also be included. The unit costs will be obtained from the Generalitat Valenciana catalogue.

Additionally, an analysis will be performed from a social perspective, including also indirect health care costs due to lost productivity of the patient (number of sick leaves and time to return to work after the event). Indirect costs will be reported by the patient in the follow-up data collection logbook. Effectiveness will be assessed by the physical activity performed by the patient, reported by the patient at the end of the follow-up, measured in METs-minutes/week.

# Quality Control

The investigators will keep the study documents with clinical follow-up value in their corresponding clinical records as they are not extraordinary procedures. All information collected in the CRFs will be cross-checked with the notes made in the clinical record. The investigator undertakes to complete the CRFs diligently and clearly. The investigator will keep a copy of the informed consent form. informed consent. The study data will be kept for 5 years according to current legislation so that it can be hypothetically reviewed at a later date.

# Monitoring

Monitoring will be carried out through telephone calls to the participating investigators as well as an on-site visit to each participating centre, with the aim of supervising the development of the study and the correct compliance with the SOPs.

The main tasks that are carried out in the monitoring process are

- Summary of the study (inclusion and exclusion criteria, objectives, main objectives of the study, special characteristics of the project).

- Advice on doubts related to the protocol.

- Reminder of Good Clinical Practice procedures.

- Review the correct obtaining of Informed Consent.

- Advice on how to fill in the DCL.

- Monitoring the inclusion of patients

- Verify that the data appearing in the clinical history and in the DCL are the same, as well as checking the appropriate annotations in the patient's clinical history.

The monitor will request the investigator's assistance to diligently carry out his or her tasks.

# Withdrawal of patients from study

The withdrawal of a patient from the study may be due to the Investigator's final decision or the patient's decision to leave the rehabilitation program.

## Criteria for definitive withdrawal from study

A patient may withdraw her consent, if she so chooses, at any time and for any reason, or it may be withdrawn by decision of the Investigator. As far as possible, care should be taken to document the reason for withdrawal and record it in the DCL as:

- At the patient's own request.

- Physician's decision.

## Procedures and Consequences of a patient’s withdrawal from the study

Patients may leave the study if they choose to do so, at any time and for any reason.

If the patient leaves the study for any reason, the patient should be evaluated using the procedure normally provided for the end-of-study visit.

For patients who do not return to the site, the Investigator should make every effort to recontact them (e.g., by contacting their family members or private physician, reviewing available records or the health database) and determine their health status, including at least their vital status. Attempts to contact them should be recorded in the patient's records (e.g. times and dates of telephone contact attempts or proof of sending registered letters).

If LDL cholesterol levels are above 100 mg/dl at 4 months, the patient will be excluded from the study for protocol reasons. If the patient is in the conventional rehabilitation group, medical treatment will be increased and he/she will be followed until the end of the study, although his/her data will not be analysed in the calculation of the results of the study. If you are in the telerehabilitation group, your medication will be increased and you will continue in the program until the end, although, as in the previous case, your data will not be considered for the statistical analysis.

The statistical analysis plan will specify how these patients will be considered.

Patients who have dropped out of the study cannot be readmitted to the study. Their inclusion number should not be used.

# Ethical Aspects

## Accordance with the regulations in force

The study will be governed at all times by the ethical principles contained in the Declaration of Helsinki and subsequent amendments.

See at: http://www.wma.net/en/30publications/10policies/b3/index.html.

The study will be governed by the principles of Good Clinical Practice (ICH Tripartite and Harmonised Guidelines for Good Clinical Practice) and therefore following the appropriate standard operating procedures.

Prior to the start of any study procedure, approval by the Clinical Research Ethics Committees of the 3 hospitals participating in the study is required.

## Benefit-risk assessment for subjects participating in the study.

Participants undergoing conventional rehabilitation and tele-rehabilitation perform a symptom-limited exercise stress test before starting physical exercise. In patients at low risk, such as the participants in this study, or even moderate risk, a symptom-limited exercise stress test is not considered imperative beforehand. In the present protocol, the stress test serves not to stratify the risk, which in principle is low in itself, but to establish the training heart rate and to serve as a comparator between groups for the functional capacity achieved after the rehabilitation program. In addition, it adds a safety factor for patients entering the two programs being evaluated.

The recommendations of the guidelines of the main Scientific Societies on equipment for the Rehabilitation Unit are followed, with personnel trained in CPR, a semi-automatic defibrillator, a crash cart and an outlet for supplying supplementary oxygen. Each hospital also has a protocol for action in the event of an emergency. Healthcare personnel are trained in the identification of symptoms of discomfort and in the use of the Börg scales. Prior to the start of each exercise session, an assessment of the patient's general condition is required, including: presence of cardiac symptoms, measurement of blood pressure, advice on medication compliance, and heart rate. Possible incidents suffered by each patient in the home activity program are also discussed.

Compared to the minimal risks of these programs, the benefit achieved in terms of reduced morbidity and mortality suggests that cardiac rehabilitation should be extended to the vast majority of patients who suffer an acute coronary event. This protocol is in fact a step forward, not only in the promotion of this new service to be offered by the Hospital Arnau de Vilanova, but also an attempt to expand, if the tele-rehabilitation strategy proves to be superior, the number of subjects who could have access to these programs.

## Information and informed consent sheet.

The study investigator shall explain to patients (or their legally authorised representatives) the nature of the study, its purposes, procedures, estimated duration, potential risks and expected benefits. It will be clearly stated that participation is voluntary and that they may withdraw from the study at any time and without detriment to their future health care. Before signing, you should read the contents and comment or ask any questions you may have. Two documents shall be signed and kept by the patient and the investigator. No procedure will be carried out unless this consent form has been signed.

## Data confidentiality

1. Regarding Investigators and personnel who have access to study procedures and data

Information provided by the Sponsor (or by any company/institution acting on its behalf), or produced during the clinical trial, including, but not limited to, the protocol, DCL, Investigator's Brochure, and results obtained during the course of the trial, will be considered confidential prior to publication of the results. The Investigator and any person under his/her authority agree to maintain the confidentiality of such information and not to share it with anyone without the prior written consent of the Sponsor.

However, the submission of this protocol and other documentation required by the IRB/IEC is expressly permitted, and IRB/IEC members are subject to the same obligations of confidentiality.

The Sub-Investigators shall be subject to the same obligations as the Investigator. The Investigator should inform the Sub-Investigators of the confidential nature of the clinical trial. The Investigator and the Sub-Investigators shall use the information provided only for the purposes specified in the clinical trial, excluding any personal use or use on behalf of a third party.

In addition, the Investigator and the Sponsor agree to adhere to the principles of confidentiality of personal data in relation to the patients, the Investigator, and the collaborators involved in the study.

1. Regarding the patients

The confidentiality of each patient's data will be respected at all times. The affiliation of each of the patients and their association with the study and, thus, access to the data collected in the DCL is ensured by a document to which only the investigators, monitors and health authorities, if applicable, have access. The DCLs contain only the identification number of each subject included. The data obtained will be handled in accordance with the Spanish regulations on the handling of computerised data (LO 15/1999, of 13 December on the protection of personal data).

## Interference with individual doctor's prescribing habits

The use of drugs is not evaluated in this study. The drugs prescribed at discharge from hospital can be modified by the various physicians doing the clinical follow-up with the aim of achieving optimal care for each pathology or improvement of cardiovascular risk factors. At discharge, the appropriate statin will be prescribed at the appropriate dose to hypothetically achieve a lowering of LDL cholesterol below 70 mg/dl according to the percentage of LDL lowering recognised for each statin and dose. During the study there will be no dose modification except for LDL levels above 100 mg/dl at 4 months, in which case the patient will be withdrawn from the study, as this variable is one of the efficacy parameters evaluated within the new telemonitoring strategy. Obviously, the patient will patient will obviously remain in the telerehabilitation program, although his or her final data will not be evaluable.

## Responsibility of all study participants.

A. Researcher

a. Sign a commitment in which they acknowledge themselves as investigators of the study and affirm that they are aware of the protocol and any modifications to it, being in agreement with its contents.

b. Inform the research subjects and obtain their consent.

c. Collect and record data correctly and respond to audits if necessary.

d. Respect data confidentiality.

e. Facilitate monitoring visits.

f. Be accountable to the scientific and professional community for the objectives, basic methodology and significance of the results of the study.

g. Keep on file:

- Copy of the authorisations of the CEIC and of the competent Health Authorities.

- Copy of the identification sheet of the selected patients.

- The original consent forms.

- A copy of the protocol.

- A copy of the CRD sheets.

- Accounting and sample receipt documents.

- Any correspondence relating to the study

All documentation should be retained for 15 years after the date of the final report. The responsibility for storage may be delegated to the sponsor by means of a certified statement. In such a form the investigator should at least retain the sheets identifying the subjects participating in the trial and the informed consent.

B. Coordinator Researcher

a. Sign the protocol and any amendments thereto together with the promoter.

b. Co-responsible with the promoter for the elaboration of the monitoring and final reports.

c. Contribute to the dissemination of the results of the study together with the promoter.

1. Monitor

The monitors will attest to the accuracy of the information collected in the CRDs and should be provided with the facilities of the investigative team.

D. Promotor

a. Sign the protocol and any amendments to the protocol with the coordinating investigator.

b. Providing the investigators with the protocol

c. Submit the protocol to the IRB/IEC

d. Request authorisation from the Administration and submit the corresponding documentation.

e. Submit the follow-up and final reports in due time and form, as well as communicate the termination or interruption, if any, and its cause.

f. Deliver a copy of the protocol and attached documentation to the heads of the health care service providers where the protocol is to be implemented.

g. Apply quality control on the data to ensure that they are reliable.

h. Identify sources of funding for the study

i. Sign the contract with the appropriate entity.

j. Make the results of the study public, if possible by publication in a scientific journal.

## Clinical Research Ethics Committee (IRB)

The protocol, consent form and other accompanying information will be reviewed by the IRB/IEC of the hospital concerned. Any modifications other than administrative changes must be approved by the IRB/IEC.

Changes in study conduct without amendment are considered violations that may lead to disqualification of the site.

## Study budget

In accordance with Royal Decree 561/93, the study will not start until the Director of the Centre where the study is being carried out has given his approval. To this end, a contract will be signed which will establish the amount of financial compensation for both the research team and the Centre in terms of direct and indirect expenses, if applicable.

## Insurance policy

With respect to any direct or indirect liability caused by investigational products in connection with this clinical trial, Nabelia assumes legal responsibility on behalf of the investigator and co-workers for any harm caused to patients by the use of the ITS system. This is on the condition that the investigators and co-workers have followed the instructions in accordance with the protocol, that the trial products have been supplied by Nabelia, and that the principal investigator and co-workers conduct the trial in accordance with the Standards of Good Clinical Practice.

TSB Technologies is in contact with various insurance companies to take out civil liability insurance. Given that the procedures to which the patients are subjected are the same as those carried out in normal clinical practice in the cardiac rehabilitation programs, the AEMPS will be asked for exemption from contracting this insurance. In the event that the application is rejected, the insurance policy will be taken out, complying with the points specified in Royal Decree 56/1993 of 16 April 1993, published in the Official State Gazette of 13 May 1993.

# Final report and dissemination of results

## Monitoring and final reports

During the study and once it is completed, the corresponding reports will be sent to the Spanish Agency for Medicines and Health Products, in accordance with the regulations in force.

## Dissemination of results

The Scientific Coordinator of the study together with the sponsor will be responsible for the timely dissemination of the results through the usual scientific means. As a multicentre study, it is mandatory that the first publication is based on data obtained from all patients analysed in the study. Collaborating investigators must agree not to submit data on an individual basis or for a subgroup of sites prior to the first full publication by the sponsor.

The sponsor will review submissions for accuracy (avoiding possible discrepancies with information submitted to the Health Authorities), verify that confidential information is not inadvertently disclosed, and provide any additional supplementary information.

# Bibliography

1. Graham I, Atar D, Borch-Johnsen K, Boysen G, Burell G, Cifkova R, Dallongeville J, De Backer G, Ebrahim S, Gjelsvik B, Herrmann-Lingen C, Hoes A, Humphries S, Knapton M, Perk J, Priori SG, Pyorala K, Reiner Z, Ruilope L, Sans-Menendez S, Scholte op Reimer WJ, Weissberg P, Wood D, Yarnell J, Zamorano JL, Walma E, Fitzgerald T, Cooney MT, Dudina A, Vahanian A, Camm J, De Caterina R, Dean V, Dickstein K, Funck-Brentano C, Filippatos G, Hellemans I, Kristensen SD, McGregor K, Sechtem U, Silber S, Tendera M, Widimsky P, Zamorano JL, Hellemans I, Altiner A, Bonora E, Durrington PN, Fagard R, Giampaoli S, Hemingway H, Hakansson J, Kjeldsen SE, Larsen ML, Mancia G, Manolis AJ, Orth-Gomer K, Pedersen T, Rayner M, Ryden L, Sammut M, Schneiderman N, Stalenhoef AF, Tokg Aˆzoglu L, Wiklund O, Zampelas

A. European guidelines on cardiovascular disease prevention in clinical practice: executive summary. Eur Heart J 2007;28:2375–2414.

1. Smith SC, Benjamin EJ, Bonow RO, Braun LT, Creager MA, Franklin BA, Gibbons RJ, Grundy SM, Hiratzka LF, Jones DW, Lloyd-Jnes DM, Minissian M, Mosca L, Peterson ED, Sacco RL,

Spertus J, Stein JH, Taubert KA. AHA/ACCF secondary prevention and risk reduction therapy for patients with coronary and other atherosclerotic vascular disease: 2011 update. Circulation 2011;124:2458–2473.

1. Kotseva K, Wood D, De Backer D, Pyorala K, Keil U,, EUROASPIRE study group,: EUROASPIRE III: a survey on the lifestyle, risk factors and use of cardioprotective drug therapies in coronary patients from 22 european countries. Eur J Cardiovasc Prev Rehabil 2009;16:121-37
2. West RR, Jones DA, Henderson AH. Rehabilitation after myocardial infarction trial (RAMIT): multicentre randomised controlled trial of comprehensive cardiac rehabilitation in patients following acute myocardial infarction. Heart 2012;98:637-44
3. West R. Dee J. Cardiac rehabilitation after myocardial infarction: is the emperor wearing his clothes? (or does cardiac rehabilitation reduce mortality?). 2013.
4. Zwisler ADO, Soja AMB, Rasmussen S, Frederiksen M, Abadini S, Appel J, Rasmussen H, Gluud C, Iversen L, Sigurd B, Madsen M, Fischer-Hansen J. Hospital-based comprehensive cardiac rehabilitation versus usual care among patients with COngestive heart failure, ischemic heart disease, or high risk of ischemic heart disease: 12-month results of a randomized clinical trial. Am Heart J 2008;155:1106–1113.
5. Giannuzzi P, Temporelli PL, Marchioli R, Maggioni AP, Balestroni G, Ceci V, Chieffo C, Gattone M, Griffo R, Schweiger C, Tavazzi L, Urbinati S, Valagussa F, Vanuzzo D. Global secondary prevention strategies to limit event recurrence after myocardial infarction: results of the gospel study, a multicenter, randomized controlled trial from the Italian cardiac rehabilitation network. Arch intern med 2008;168:2194–2204.
6. Taylor RS, Brown A, Ebrahim S, Jolliffe J, Noorani H, Rees K, Skidmore B, Stone JA, Thompson DR, Oldridge N. Exercise-based rehabilitation for patients with coronary heart disease: systematic review and meta-analysis of randomized controlled trials. Am J Med 2004;116:682–692.
7. Taylor RS, Unal B, Critchley JA, Capewell S. Mortality reductions in patients receiving exercise-based cardiac rehabilitation: how much can be attributed to cardiovascular risk factor improvements? Eur J Cardiovasc Prev Rehabil 2006;13:369–374.
8. Martin BJ, Hauer T, Arena R, Austford LD, Galbraith PD, Lewin AM, Knudtson ML, Ghali WA, Stone JA, Aggarwal SG. Cardiac rehabilitation attendance and outcomes in coronary artery disease patients/clinical perspective. Circulation 2012;126:677–687.
9. Suaya JA, Stason WB, Ades PA, Normand SL, Shepard DS. Cardiac rehabilitation and survival in older coronary patients. J Am Coll Cardiol 2009;54:25–33.
10. Hammill BG, Curtis LH, Schulman KA, Whellan DJ, Relationship between cardiac rehabilitation and long-term risks of death and myocardial infarction among elderly Medicare beneficiaries. Circulation 2010;121:63-70.
11. Leung YW, Grewal K, Gravely-Witte S, Suskin N Stewart DE, Grace SL, Quality of life following participation in cardiac rehabilitation programs of longer of shorter than 6 months, does the duration matter?. Popul Health Manag 2011;14:181-8
12. Wen CP, Wai JP, Tsai MK, Yang YC, Cheng TY, Lee MC, Chan HT, Tsao CK, Tsai SP, Wu X. Minimum amount of physical activity for reduced mortality and extended life expectancy: a prospective cohort study. Lancet 2011;378:1244–1253.
13. Sattelmair J, Pertman J, Ding EL, Kohl HW, Haskell W, Lee IM. Dose response between physical activity and risk of coronary heart disease / clinical perspective. Circulation 2011;124:789–795.
14. Onishi T, Shimada K, Sato H, et al. Effects of phase III cardiac rehabilitation on mortality and cardiovascular events in elderly patients with stable coronary artery disease. *Circ J* 2010;74:709-714.
15. Franklin BA, Hall L, Timmis GC. Contemporary cardiac rehabilitation services. *Am J Cardiol*

1997;79:1075-1077.

1. DeBusk RF, Houston Miller N, Superko HR, et al. A case-management system for coronary risk factor modification after acute myocardial infarction. *Ann Intern Med* 1994;120:721-729.
2. Carlson JJ, Johnson JA, Franklin BA, Vanderlaan RL. Program participation, exercise adherence, cardiovascular outcomes, and program cost of traditional versus modified cardiac rehabilitation. *Am J Cardiol* 2000;86:17-23.
3. Gomis M, Gil D, Lopez L, et al. Impact of mHealth in heart transplant management (mHeart). *IJIC* 2016; 16(6): A38

# ANNEXES

**Annex 1: Data collection notebook**

**Annex 2: Agreement of the Head of Service**

**Annex 3: Commitment of the principal investigator**

**Annex 4: Commitment of the collaborating investigator**

**Annex 5: Patient informed consent**

## ANEX 1.

**VISIT - 1 // PRESELECTION**

| Patient Initials |  | | |
| --- | --- | --- | --- |
| Patient identification Code (n) |  | | |
| Age (years) |  | Gender (M/F) |  |
| Date (DD-MM-YY) |  | | |

**PRESELECTION CRITERIA**

| Age < 72 years (Y/N) |  |
| --- | --- |
| NSTEACS, STEACS or unstable angina (Y/N) |  |

**EXCLUSION CRITERIA**

| Uncontrolled atrial or ventricular arrhythmias (Y/N) |  |
| --- | --- |
| Valvopathy of at least moderate grade (Y/N) |  |
| Heart failure NYHA 2/4 or above (Y/N) |  |
| Malignancy or major cardiovascular, pulmonary, renal or hepatic disease as judged by the investigator (Y/N) |  |
| Cerebrovascular disease, such as cerebral ischaemia, infarction, embolism or haemorrhage (Y/N) |  |
| Uncontrolled HTA Y/N) |  |
| Moderate pulmonary hypertension (Y/N) |  |
| Thrombophlebitis (Y/N) |  |
| Uncontrolled endocrine pathology (Y/N) |  |
| Limiting musculoskeletal pathology (Y/N) |  |
| Psychiatric pathology (Y/N) |  |
| Ventricular aneurysm (Y/N) |  |
| Ejection fraction less than 50%. (Y/N) |  |

| Accepts Stress Test (Y/N) |  |
| --- | --- |
| Accepts participation (Y/N) |  |

**VISIT 0 // SELECTION**

| Patient initials |  |
| --- | --- |
| Patient identification code (n) |  |
| Date (DD-MM-YY) |  |

**SELECTION CRITERIA**

| Symptom-limited stress test without angina or electrical ischaemia (Y/N) |  |
| --- | --- |
| Informed consent (Y/N) |  |

**VISIT 1 // RANDOMISATION**

**PERSONAL DATA**

| Patient initials |  |
| --- | --- |
| Patient identification code (n) |  |
| Randomisation number (n) |  |
| Date (DD-MM-AA) |  |

**CLINICAL DATA**

| Unstable angina (Y/N) |  |
| --- | --- |
| NSTEACS (Y/N) |  |
| STEACS (Y/N) |  |
| Number of vessels (n) |  |
| Number of Stents (n) |  |
| Bypass Number (n) |  |
| Complete revascularisation (Y/N) |  |
| Diabetes mellitus (Y/N) |  |
| Arterial hypertension (Y/N) |  |
| Dyslipidaemia (Y/N) |  |
| Active smoking (Y/N) |  |
| Ex-smoker (Y/N) |  |
| Ejection fraction (%) |  |

**ADITIONAL MEDICATION**

| Beta-blockers (Y/N) |  |
| --- | --- |
| Calcium antagonists (Y/N) |  |
| Nitrates (Y/N) |  |
| Anti-aggregants (Y/N) |  |
| ACE inhibitors / ARBs (Y/N) |  |
| Lipid lowering agents (Y/N) |  |
| Others (Y/N) |  |

**PHYSICAL EXAMINATION**

| Height (cm) |  | Weight (Kg) |  |  |  |
| --- | --- | --- | --- | --- | --- |
| Heart Rate (bpm) |  | SBP (mmHg) |  | DBP (mmHg) |  |
| BMI (kg/m2) |  | Waist circumference (cm) |  | Hip circumference (cm) |  |

**STRESS TEST**

| VO2max |  |
| --- | --- |
| Effort time (min) |  |
| Maximum heart rate (Bpm) |  |
| Maximum blood pressure (mmHg) |  |
| METS (n) |  |
| ST descent (Y/N) |  |
| Angor (Y/N) |  |

**BLOOD TESTS**

| Glucose (mg/dl) |  | Creatinine (mg/dl) |  |
| --- | --- | --- | --- |
| Total cholesterol (mg/dl) |  | HDL cholesterol (mg/dl) |  |
| LDL cholesterol (mg/dl) |  | Triglycerides (mg/dl) |  |
| CRPus (mg/l) |  | HgA1c (%) |  |

**ARTERIAL STIFFNESS**

PWV (m/s)

**SURVEYS**

| IPAQ (n) |  |
| --- | --- |
| PREDIMED (n) |  |
| HAD ANXIETY (n) |  |
| HAD DEPRESSION (n) |  |
| EQ-5D |  |
| FAGERSTRÖM (n) |  |

**CLINICAL EVENTS**

| Consultation in the emergency department (Y/N) |  |
| --- | --- |
| Hospitalised (Y/N) |  |
| Revascularisation (Y/N) |  |

**SOCIO-OCCUPATIONAL SITUATION**

| Active worker until the event (Y/N) |  |
| --- | --- |

**VISIT 2 // MONTH 4**

**FILIATION DATA**

| Patient initials |  |
| --- | --- |
| Patient identification code (n) |  |
| Randomisation number (n) |  |
| Date (DD-MM-YY) |  |

**PHYSICAL EXAMINATION**

| Height (cm) |  | Weight (Kg) |  |  |  |
| --- | --- | --- | --- | --- | --- |
| Heart rate (bpm) |  | SBP (mmHg) |  | DBP (mmHg) |  |
| BMI (kg/m2) |  | Waist circumference (cm) |  | Hip circumference (cm) |  |

**BLOOD TESTS**

| Glucose (mg/dl) |  | Creatinine (mg/dl) |  |
| --- | --- | --- | --- |
| Total cholesterol (mg/dl) |  | HDL cholesterol (mg/dl) |  |
| LDL cholesterol (mg/dl) |  | Triglycerides (mg/dl) |  |
| CRPus (mg/l) |  | HgA1c (%) |  |

**CLINICAL EVENTS**

| Consultation in the emergency department (Y/N) |  |
| --- | --- |
| Hospitalised (Y/N) |  |
| Revascularisation (Y/N) |  |

**SOCIO-OCCUPATIONAL SITUATION**

| Active worker (Y/N) |  |
| --- | --- |
| Time to return to work after discharge (días) |  |

**VISIT 3 // MONTH 10**

**FILIATION DATA**

| Patient initials |  |
| --- | --- |
| Patient identification code (n) |  |
| Randomisation number (n) |  |
| Date (DD-MM-YY) |  |

**PHYSICAL EXAMINATION**

| Height (cm) |  | Weight (Kg) |  |  |  |
| --- | --- | --- | --- | --- | --- |
| Heart rate (bpm) |  | SBP (mmHg) |  | DBP (mmHg) |  |
| BMI (kg/m2) |  | Waist circumference (cm) |  | Hip circumference (cm) |  |

**BLOOD TESTS**

| Glucose (mg/dl) |  | Creatinine (mg/dl) |  |
| --- | --- | --- | --- |
| Total cholesterol (mg/dl) |  | HDL cholesterol (mg/dl) |  |
| LDL cholesterol (mg/dl) |  | Triglycerides (mg/dl) |  |
| CRPus (mg/l) |  | HgA1c (%) |  |

**STRESS TEST**

| VO2max |  |
| --- | --- |
| Effort time (min) |  |
| Maximum heart rate (Bpm) |  |
| Maximum blood pressure (mmHg) |  |
| METS (n) |  |
| ST descent (Y/N) |  |
| Angor (Y/N) |  |

**ARTERIAL STIFFNESS**

PWV (m/s)

**SURVEYS**

| IPAQ (n) |  |
| --- | --- |
| PREDIMED (n) |  |
| HAD ANXIETY (n) |  |
| HAD DEPRESSION (n) |  |
| EQ-5D |  |
| FAGERSTRÖM (n) |  |

**CLINICAL EVENTS**

| Consultation in the emergency department (Y/N) |  |
| --- | --- |
| Hospitalised (Y/N) |  |
| Revascularisation (Y/N) |  |

**SOCIO-OCCUPATIONAL SITUATION**

| Active worker (Y/N) |  |
| --- | --- |
| Time to return to work after discharge (días) |  |

## ANEX 2.

**Conformity of the Head of Service**

Dr. , as Head of Service of

Cardiology of the Hospital Universitario Arnau de Vilanova de Valencia,

I hereby declare:

- That I am aware of the documentation relating to the study Prevention and rehabilitation after acute coronary syndrome: new strategy through telemonitoring, whose protocol code is TSB2014.

- That the principal investigator is Dr. Ernesto Dalli Peydró.

- That the principal investigator and the rest of the staff of the team have the characteristics and are competent to carry out this study.

- That the Service and the Hospital have the resources and resources to carry out the aforementioned study.

- That I agree to carry out the study.

In Valencia, the………………………of……………………………..of 2020

Signed:

Head of Cardiology Department

Hospital Arnau de Vilanova. Valencia

## ANEX 3.

**Commitment of the principal investigator**

Dr. , as principal investigator

principal investigator I hereby declare,

- That I am aware of and agree to participate as principal investigator in the clinical trial entitled: **Prevention and rehabilitation after acute coronary syndrome: new strategy through telemonitoring.** Protocol code TSB2014 and version date: V.5 of 30 March 2020.

- That I undertake that each subject will be treated and monitored in accordance with the protocol authorised by the Clinical Research Ethics Committee and the Spanish Medicines Agency, guaranteeing the authenticity of the data obtained.

- That I will respect the ethical rules applicable to this type of study.

- That I have the material and human resources to carry out this type of study.

- That for the purposes of Organic Law 15/1999 of December on the Protection of Personal Data, I authorise my personal data to be included in a file owned by Nabelia, for the purpose of normal monitoring of the study and I agree to sign the necessary documents to formalise this authorisation.

- That I guarantee that the study monitor will have access to the clinical data of the patients included in the study after signing the corresponding confidentiality agreements.

Signed: Dr.

In Valencia, the………………………of……………………………..of 2020

## ANEX 4.

**Commitment of collaborating researchers**

Dr. , as a collaborating

as a collaborating researcher, I hereby declare,

- That I am aware of and agree to participate as a collaborating researcher in the clinical trial entitled: **Prevention and rehabilitation after acute coronary syndrome: new strategy through telemonitoring.** Protocol code: TSB2014 and version date: V.5 of 30 March 2020.

- That I undertake that each subject will be treated and monitored in accordance with the protocol authorised by the Clinical Research Ethics Committee and the Spanish Agency for Medicines and Health Products, guaranteeing the authenticity of the data obtained.

- That I will facilitate the monitor's access to the clinical data of the patients after signing the corresponding confidentiality agreement.

- That I will respect the ethical rules applicable to this type of study.

Signed: Dr.

In Valencia, the………………………of……………………………..of 2020

## ANEX 5.

**PATIENT INFORMATION SHEET**

STUDY TITLE: **CARDIOVASCULAR REHABILITATION AFTER ACUTE CORONARY SYNDROME: A NEW STRATEGY USING TELEMONITORING**

Study code: TSB2014

Date: V.5 of 30 March 2020

Sponsor: Catcronic Salut, S.L. Nabelia

This document contains information that you should know before giving your consent to participate in a clinical trial evaluating two cardiovascular rehabilitation strategies.

WHAT IS CARDIOVASCULAR REHABILITATION FOR AND WHAT DOES IT CONSIST OF?

Cardiovascular rehabilitation after acute coronary syndrome or unstable angina with or without percutaneous or surgical revascularisation is indicated by the major scientific societies as a class I indication, i.e. there is evidence that it improves quality of life and reduces complications and mortality by 25%, with a level of evidence A, i.e. following the results of large population studies. Cardiovascular rehabilitation is a therapeutic process whose purpose is to achieve functional recovery and a return to normal activities prior to the cardiac event. Strengthening is achieved through physical exercise, as well as the adoption of a series of psychological guidelines and healthy lifestyle habits for the control of the so-called risk factors, mainly tobacco, high blood pressure, high cholesterol, etc.

The aim of this non-drug clinical trial is to evaluate two types of cardiovascular rehabilitation in relation to the maintenance of physical activity, improvement of quality of life, cardiovascular health parameters and cost-effectiveness analysis.

HOW IT IS DONE.

Before proposing you to participate in the study, the physician will conduct a thorough review of your medical history to ensure that you are at low overall risk and will propose a symptom-limited exercise test at least 7 days after discharge from hospital. If you perform this stress test without any alterations and agree to participate in the rehabilitation program, you will be randomly included in one of the two rehabilitation programs to be compared and described below.

One program is called CONVENTIONAL REHABILITATION. It consists of coming to the hospital for 2 months, twice a week (16 visits), for 8 sessions of physical training and 8 educational talks to promote psychological and cardiovascular health. The physical training consists of warm-up exercises for about 10 minutes, an exercise wheel for 30 minutes and finally recovery for 10 minutes. Blood pressure, heart rate and oxygen saturation are monitored, always under the supervision of health personnel. If during the exercise you notice any symptoms such as pain, shortness of breath or dizziness, notify the doctor or nurse. On the days that you do not go to the hospital, you will have to walk progressively longer distances. At the end of the 2 month period, you will continue to be cared for by your family doctor and/or your usual specialist.

The other program is called TELE-REHABILITATION. It consists of coming to the hospital for 2 weeks, twice a week (4 visits), to carry out 3 physical training sessions and attend 6 educational talks on psychological and cardiovascular health. The physical training will consist of a 10-minute warm-up, 30 minutes of walking and 10 minutes of recovery. Blood pressure, heart rate and oxygen saturation are monitored, always under the supervision of health personnel. If during the exercise you notice any symptoms such as pain, shortness of breath or dizziness, please notify the doctor or nurse. From the first day you will be given a mobile phone with an application that will guide you during your exercise sessions, both in the hospital and later at home. Together with the phone application, you will be given access to an internet address that will complement the monitoring of physical activity with recommendations, messages and information with the aim of better compliance with cardiovascular prevention objectives. This program will run for 10 months. Regardless of participation in the study, you will attend regular check-ups with your general practitioner and cardiologist. The conventional rehabilitation program will be carried out at the Hospital Universitario La Fe and the tele-rehabilitation program at the Hospital Universitario Arnau de Vilanova. All participants will undergo the same assessments, which include a treadmill stress test with measurement of oxygen consumption before starting the program and at the end of the program. Blood tests at baseline, 4 months and 10 months. Study-specific medical visit including 4 surveys, at the start of the study, at 4 months and at 10 months. The stress test, despite being a safe test, with a mortality rate of 1 per 10,000 and a morbidity rate of 2 per 10,000, it entails certain risks, complications that can be classified, according to their severity, into minor and major. The minor ones are transient arterial hypertension, supraventricular tachycardia, heart failure, chronotropic failure, extrasystoles. The major ones are ventricular tachycardia, myocardial infarction, syncope and ventricular fibrillation and death.

WHAT BENEFITS ARE EXPECTED TO BE ACHIEVED

Physical exercise, practised regularly, improves your quality of life. It reduces the sensation of suffocation and tiredness, improves blood pressure and glycaemia control, facilitates weight control, greater tolerance to carry out daily life and work activities, improves mood and quality of life. Helps control risk factors, reduces progression of coronary lesions, improves collateral circulation, reduces risk. Helps combat anxiety and depression and increases enthusiasm and optimism. Improves osteoporosis. It helps with sleep and decreases the risk of death and increases life expectancy. With either program you are expected to gain these benefits.

WHAT ARE THE RISKS

You perform a symptom-limited stress test to minimise the occurrence of future complications during physical exercise as well as to program your training heart rate to a safe level. However, any medical action has risks. The most frequent symptoms or possible adverse effects of exercise are tiredness, increased blood pressure (in people with poor blood pressure control), dizziness, hypoglycaemic crisis (poorly controlled diabetics), non-specific chest pain, angina pectoris, mild arrhythmic disturbances (isolated supra- or ventricular extrasystoles), bronchospasm crises or increased shortness of breath, if you perform strenuous or sudden exercise, increased joint pain or tendon inflammation due to overload, if you have previous injuries. If you have arterial blockage in the lower limbs, you will experience pain during exercise that will disappear with rest. The most serious, especially if the exercise is sudden and intense, can be syncope, severe rhythm disturbances (ventricular fibrillation), heart failure or severe bronchoconstriction crisis with difficulty in breathing. The risk of sudden death is exceptional. Myocardial infarction and cardio-respiratory arrest is very rare. Other rare complications of sudden exercise include rhabdomyolysis (damage to muscle tissue with generalised decompensation and renal failure), hyperthermia (severe temperature increase) or hypothermia (temperature decrease), dehydration, urticaria and even anaphylaxis (severe allergic reaction of the whole body). To avoid these complications, a proper risk assessment and risk stratification is performed before starting the training program. In your current clinical condition, the benefits of this treatment outweigh the possible risks. For this reason, you are advised to undergo this treatment. To minimise the risks, it is absolutely essential that you inform the program professional (doctor, nurse, physiotherapist) of any abnormalities (pain, dizziness, cold sweats, general malaise, etc.) that you may have experienced the previous day. During the exercise session, you must inform the health staff of any abnormality you present. Follow the rules described in the documents provided.

OTHER ALTERNATIVES AVAILABLE FOR YOU

The Clinical Practice Guidelines for cardiac rehabilitation recommend this type of treatment because of its proven scientific evidence (see benefits section). If you do not agree to participate in the rehabilitation program, the alternative is unsupervised exercise.

PROCEDURES AND CONFIDENTIALITY

This clinical trial will involve 60 people like yourself who have just had an acute coronary event. Of these, 30 will undergo the conventional rehabilitation program and 30 the telerehabilitation program. 30 will do the telerehabilitation program. If you agree to participate in the study, no extraordinary procedures will be performed that would be performed if you were following the rehabilitation program established in your hospital. If you agree to participate, some of your clinical data will be known to the health care staff attending you, the research ethics committee and the health authorities. Your personal affiliation data will not be included in any of the study documents. The information collected cannot be associated with an identifiable person. During the study, you are guaranteed strict compliance with Organic Law 3/2018, of 5 December, on the Protection of Personal Data and guarantee of digital rights.

As of 25 May 2018, the new EU legislation on personal data, specifically Regulation (EU) 2016/679 of the European Parliament and of the Council of 27 April 2016 on Data Protection (GDPR), is fully applicable. It is therefore important that you are aware of the following information:

-In addition to the rights you already know (access, modification, opposition and cancellation of data) you can now also limit the processing of data that are incorrect, request a copy or that the data you have provided for the study be transferred to a third party (portability). To exercise your rights, please contact the principal investigator of the study: Dr. Ernesto Dalli Peydró: [dalli_ern@gva.es](mailto:dalli_ern@gva.es) We remind you that the data cannot be deleted even if you stop participating in the trial in order to ensure the validity of the research and to comply with legal obligations and drug authorisation requirements. You also have the right to contact the Data Protection Agency if you are not satisfied.

- Both the Centre and the Sponsor are respectively responsible for the processing of your data and undertake to comply with the data protection regulations in force. The data collected for the study will be identified by a code, so that no information that can identify you is included, and only your study doctor/collaborators will be able to relate this data to you and your medical history. Therefore, your identity will not be disclosed to any other person except to health authorities, when required or in cases of medical emergency. Research Ethics Committees, representatives of the Health Inspection Authority and personnel authorised by the Sponsor may only have access to verify personal data, clinical trial procedures and compliance with the standards of good clinical practice (while maintaining the confidentiality of the information).

- The Investigator and the Sponsor are obliged to retain the data collected for the study for at least 25 years after completion of the study. Thereafter, your personal information will only be retained by the Facility for your health care and by the Sponsor for other scientific research purposes if you have given your consent to do so, and if permitted by applicable law and ethical requirements.

- If we transfer your encrypted data outside the EU to our group entities, service providers or collaborating scientific researchers, the participant's data will be protected with safeguards such as contracts or other mechanisms by data protection authorities. If the participant would like to know more about this, please contact the Data Protection Officer of the Study Sponsor: Alicia Damon Estrella: [adamon@safe-lopd.com](mailto:adamon@safe-lopd.com) You may exercise your right to access, rectify or delete your data by contacting the principal investigator of the study.

WITHDRAWAL FROM THE STUDY

Your participation is completely voluntary and you can withdraw your consent at any time without this affecting your health care.

This clinical trial is financially endowed with a budget provided by the company Nabelia for the acquisition of the technology and elements that are delivered to the participants in the tele-rehabilitation program, including mobile phones with internet access, as well as T-shirts and cardiac electrical activity sensors, for the duration of the study only. Although the procedures carried out are exactly the same as the usual clinical practice in each of the hospitals, this clinical trial is covered by liability insurance, in accordance with the current regulations contracted with the Markel International Insurance Company LTD, which applies to this type of study.

Before giving your consent, you may obtain any additional information you wish from your doctor.

Dr._____________________________________Contact telephone number:______________

Informed Consent

Mr/Ms._________________________________________________________________ , I have been informed by Dr.____________________________________________ and I declare that:

- I have read the background information,

- I have had the usefulness of both cardiac rehabilitation programs explained to me,

- I have received satisfactory answers to the questions raised,

- That no extraordinary and invasive examinations are carried out,

- I understand that my participation is voluntary,

- I understand that my data will be treated confidentially,

- I understand that I can withdraw from the study at any time, without having to give explanations and without affecting my medical care.

-I consent to the scientific treatment of the analytical data obtained.

I voluntarily give my consent for the aforementioned procedure to be carried out with the means and conditions existing in the Cardiac Rehabilitation Units of the Hospital Universitario La Fe and Hospital Universitario Arnau de Vilanova in Valencia.

Patient Signature Date

Witness Signature Date

(if patient gives oral consent,

not signed)

Researcher Signature Date
