# Supplementary material for: A randomized controlled clinical trial of cardiac telerehabilitation with a prolonged mobile care monitoring strategy after an acute coronary syndrome
Source: Clin Cardiol. 2021 Dec 24;45(1):31–41. doi: 10.1002/clc.23757 (PMC8799046; doi:10.1002/clc.23757)
Supplement: Supplementary file 2 — Supporting information. [file CLC-45-31-s003.docx]

**CONSORT-EHEALTH Checklist V1.6.2 Report**

**Date completed**

03/08/2021

**by**

Ernesto Dalli

TITLE

Randomized controlled clinical trial of cardiac telerehabilitation with a prolonged mobile care monitoring strategy after an acute coronary syndrome.

**1a-i) Identify the mode of delivery in the title.**

Requested information is included in the Title.

“Randomized controlled clinical trial of cardiac telerehabilitation with a prolonged mobile care monitoring strategy after an acute coronary syndrome.”**.**

**1a-ii) Non-web-based components or important co-interventions in title**

Requested information is included in the Title.

" Randomized controlled clinical trial of cardiac telerehabilitation with a prolonged mobile care monitoring strategy after an acute coronary syndrome"

**1a-iii) Primary condition or target group in the title**

Requested information is included in the Title.

"Randomized controlled clinical trial of cardiac telerehabilitation with a prolonged mobile care monitoring strategy after an acute coronary syndrome"

ABSTRACT

**1b-i) Key features/functionalities/components of the intervention and comparator in the METHODS section of the ABSTRACT**

“…cardiac telerehabilitation (CTR), 4 in-hospital sessions and a 10-month telemonitoring, or CBCR (16 sessions)”.

**1b-ii) Level of human involvement in the METHODS section of the ABSTRACT**

“We included 67 low-risk acute coronary patients in a randomized controlled trial allocated 1:1 "..…to both interventions.

**1b-iii) Open vs. closed, web-based (self-assessment) vs. face-to-face assessments in the METHODS section of the ABSTRACT**

“Patients underwent ergoespirometry, blood test, anthropometric measurements, IPAQ, PREDIMED, HADS and EQ-5D surveys, at baseline and 10 months. Data collector was blinded to treatment assignment.”

**1b-iv) RESULTS section in abstract must contain use data**

“The primary outcome showed increased physical activity according to IPAQ survey in the CTR group with a median increase of 1726 METS-min/week *vs*. 636 in CBCR group, p=0.045. VO2max increased from baseline in the CTR group 1.7 ml/kg/min (95%CI 0.56-2.69, p<0.004), and in the CBCR group 0.6 ml/kg/min (p=0.39). ApoB/ApoA1 ratio decreased in the CTR group -0.13 (95%CI 0.03-0.24, p=0.017), without significant change in CBCR group (p=0.092). Non-HDL cholesterol did not change significantly in the CTR group (p=0.080), but increased a median of 7.3 mg/dL (IQR -2.4-18.55, p=0.021) in the CBCR group. Adherence to the Mediterranean diet**,** global well-being and quality of life improved in the CTR group and did not change in the CBCR group. Time to return to work was reduced with this telerehabilitation strategy.”

**1b-v) CONCLUSIONS/DISCUSSION in abstract for negative trials**

This is a non-negative study.

“The designed comprehensive telerehabilitation system allows minimal in-hospital training and prolonged follow-up. This strategy shows better results than CBCR."

INTRODUCTION

**2a-i) Problem and the type of system/solution**

“Paradoxically, despite the proven benefit of cardiac rehabilitation, attendance rate to these programs is a scanty 34%, according to the EUROSPIRE V registry^6^. This situation has become worse as a result of hospital program interruptions due to the COVID-19 pandemic^7^. Home-based cardiac rehabilitation programs closely linked with internet-based technology and mobile phone use could overcome these barriers^8^. Clinical studies comparing telemonitored outpatient programs and inpatient care programs have shown that the former is at least equally beneficial and cost-effective^9,10,11^”

**2a-ii) Scientific background, rationale: What is known about the (type of) system.**

“Clinical studies comparing telemonitored outpatient programs and inpatient care programs have shown that the former is at least equally beneficial and cost-effective^9,10,11^. It is also known that the duration of any rehabilitation program is directly proportional to the outcome achieved^12^.

With all these premises, we developed a cardiac telerehabilitation system called *Cardioplan*, which consists of a web platform and a smartphone application for monitoring diet, vital signs, exercise and medical treatment of post-ACS patients that allows, with little hospital training, a prolonged telemonitoring follow-up.”

METHODS

**3a) CONSORT: Description of trial design (such as parallel, factorial) including allocation ratio**

Study design section:

“This is a randomized controlled trial, with an intervention group that followed a cardiac telerehabilitation (CTR) programme in the Arnau de Vilanova Hospital, and a control group with centre-based cardiac rehabilitation (CBCR) programme in La Fe University Hospital.

ACS-patients from both hospitals were asked to participate at discharge. Patients were randomly assigned (1:1) to either CTR or CBCR regardless of the hospital of origin.”

**3b) CONSORT: Important changes to methods after trial commencement (such as eligibility criteria), with reasons**

Study design section:

“Before recruitment, two amendments were accepted: a third group without cardiac rehabilitation was eliminated as it was not considered ethical nor necessary, and maximal oxygen uptake (VO2max) was included as the main secondary outcome.”

**3b-i) Bug fixes, Downtimes, Content Changes**

No major changes were made during the trial. Data collection procedures or system software were not modified. The staff was the same during the whole study. (These data are not mentioned in the text)

Study population section:

” Patients were recruited after an acute coronary syndrome (unstable angina, NSTEACS or NSTEACS) between 28 May 2019 and 10 March 2020, when recruitment was stopped due to the COVID-19 pandemic”

Discussion section:

“The longest time to starting rehabilitation in the CBCR group was partly due to unforeseen delay in some patients in the scheduled visit to a rehabilitation physician. Its not excluded that it may have led to higher dropout in the CBCR group.”

The study was fortunately conducted despite the period of lockdown in Spain from 15 March to 21 June 2020. Non-essential hospital admissions were prohibited and the last three patients in the control group actually undertook non-face-to-face rehabilitation”

**4a) CONSORT: Eligibility criteria for participants**

Study population section:

“All patients underwent a symptom-limited CPET about 12 days after hospital discharge. Patient’s age was limited to 18-72 years old. All included patients had to meet low-risk criteria (no angina, ischaemic ECG changes, or severe arrhythmias on the exercise stress test), LVEF ≥ 50%, and minimum smartphone usage skills. The main exclusion criteria were reduced mobility, pulmonary diseases, neoplasms, or cognitive impairment”.

**4a-i) Computer / Internet literacy**

The requirements for participation in the study included:

….” minimum smartphone usage skills”.

**4a-ii) Open vs. closed, web-based vs. face-to-face assessments:**

Study population section:

" Patients were recruited, by face-to face assessment, at discharge after an acute coronary syndrome (unstable angina, NSTEACS or NSTEACS)."

**4a-iii) Information giving during recruitment**

Study design:

" Each participant signed an informed consent prior to participation. "

**4b) CONSORT: Settings and locations where the data were collected**

Questionnaires:

“All patients answered questionnaires at baseline and 10 months. Due to small sample size, questionnaires were administered through computer assisted face-to-face interviewing, in order to obtain higher response rates. The interviewer didn´t know patient assignation”

**4b-i) Report if outcomes were (self-)assessed through online questionnaires.**

Sef-reported adherence to Mediteranean diet (PREDIMED questionnaire), self-reported global well-being (HADS questionaire), self-reported physical activity by IPAQ questionnaire were assessed with computer assistance offline. Self-reported quality of life as measured by the EQ-5D questionnaire was assessed using printed sheets.

**4b-ii) Report how institutional affiliations are displayed**

All staff involved identified themselves beforehand.

The app displays a screen with the name of the institution and the names of all involved research staff.

**5) CONSORT: Describe the interventions for each group with sufficient details to allow replication, including how and when they were actually**

**Administered**

Control and experimental rehabilitation procedures:

“Both groups underwent the same educational sessions. During follow-up, all patients were instructed to engage in moderate physical activity at least 150 minutes a week guided by the Borg’s rating of perceived exertion scale of 12-14 (6-20 scale), as well as strength exercises twice a week”

Control group

“CBCR comprised 16 sessions (2 month) of supervised exercise delivered by a physiotherapist and a trained nurse. Physical activity consisted of a workout routine and aerobic cycling training. Warm-up and stretching exercises were included”.

Telerehabilitation group

“Hospital training comprised 4 sessions (2 weeks) with assistance of a physiotherapist and a trained nurse. Physical activity consisted of walking down a corridor, adjusting their pace to the target heart rate (60-80% of heart rate reserve), with the use of their smartphone and heart rate monitor chest strap (Polar H7, Kempele, Finland). Warm-up, stretching and resistance band exercises were also included. The smartphone app guided them through a daily schedule for 10 months. All entered data were saved on the web platform.”

**5-i) Mention names, credential, affiliations of the developers, sponsors, and owners**

**5-ii) Describe the history/development process**

This project is the result of collaboration with a technological company (Trilema Salud, Valencia, Spain) with experience in healthcare developments. The monitoring web platform has been modified and a specific app has been designed for secondary prevention and comprehensive cardiac rehabilitation.

**5-iii) Revisions and updating**

No changes have been made in the system during the trial.

**5-iv) Quality assurance methods**

The adaptation of the monitoring website and mobile app has been fully validated by cardiologists to ensure the reliability and quality of the data entered by patients.

The semi-automated text messages have been written by a cardiologist.

The use of the mobile app has been evaluated by the patients themselves anonymously as mentioned in the article.

**5-v) Ensure replicability by publishing the source code, and/or providing screenshots/screen-capture video, and/or providing flowcharts of the algorithms used**

This is the Procedures chart that was included in the protocol

|  | **Start** | **4 months** | **10 months** |
| --- | --- | --- | --- |
| **INFORMED CONSENT** | X |  |  |
| **FILIATION DATA** | X | X | **X** |
| **CLINICAL DATA** | X |  |  |
| **SOCIO-OCCUPATIONAL DATA** | X | X | X |
| **PHYSICAL EXPLORATION** | X |  |  |
| **BLOOD TEST** | X | X | X |
| **STRESS TEST** | X |  | X |
| **PHYSICAL WORKING CAPACITY** | X |  | X |
| **SURVEY** | X |  | X |
| **CLINICAL EVENTS** | X | X | X |
| **COST-EFFECTIVENESS ANALYSIS** |  |  | X |

List of items: informed consent, personal details, clinical data, socio-occupational data, physical examination, blood test, treadmill test, PWV, questionnaires, clinical events, cost-effectiveness analysis.

**5-vi) Digital preservation**

Restricted URL access is still open to healthcare staff. After completion of the study period, long term monitoring is currently in progress.

**5-vii) Access**

Access to the application is password protected. The use of the App is free of charge for patients.

**5-viii) Mode of delivery, features/functionalities/components of the intervention and comparator, and the theoretical framework**

“Control and experimental rehabilitation procedures:

Both groups underwent the same educational sessions. During follow-up, all patients were instructed to engage in moderate physical activity at least 150 minutes a week guided by the Borg’s rating of perceived exertion scale of 12-14 (6-20 scale), as well as strength exercises twice a week.

Control group

CBCR comprised 16 sessions (2 month) of supervised exercise delivered by a physiotherapist and a trained nurse. Physical activity consisted of a workout routine and aerobic cycling training. Warm-up and stretching exercises were included. “

Telerehabilitation group

Hospital training comprised 4 sessions (2 weeks) with assistance of a physiotherapist and a trained nurse. Physical activity consisted of walking down a corridor, adjusting their pace to the target heart rate (60-80% of heart rate reserve based on baseline treadmill test), with the use of their smartphone and heart rate monitor chest strap (Polar H7, Kempele, Finland). Warm-up, stretching and resistance band exercises were also included. The smartphone app guided them through a daily schedule for 10 months. All entered data were saved on the web platform.”

“Description of the comprehensive monitoring system

The Cardioplan system, designed in cooperation with Trilema Salud (Valencia, Spain), consists of the following elements:

1. A webpage to personalize healthcare and track adherence to recommendations with password-protected access. There were 7 variables monitored using a traffic light colour code. The health care team monitors the colour of the icons weekly and can communicate with patients by messaging or videoconferencing.

2. Smartphone application. (RC: Cardiac Rehabilitation), with daily scheduling of exercise sessions, record of general condition on a scale from 1 to 10, recording of vital signs, brief survey of recommended foods and checking of medication intake. The exercise module allows you to track and record every session. A smoking cessation aid module is also included. It gives access to warm-up and stretching videos, to a virtual educational classroom, and suggested websites. In case of not having a suitable device, a terminal with internet access was provided. Access is password protected. Technical assistance in case of sensor/system failure is provided through a call centre.

3. Synchronization with different heart rate monitors via Bluetooth.”

**5-ix) Describe use parameters**

The follow-up instructions for each patient are entered on the web portal. The frequency and time of day at which the requested data must be entered is scheduled. It includes the different parameters evaluated, measurements, medication, exercise sessions and surveys.

A chest strap-type heart rate sensor, linked to the mobile phone, was provided to keep the heart rate during exercise in the target range appropriate for each patient.

**5-x) Clarify the level of human involvement**

Section Telerehabilitation group:

“Data entered by the patients were monitored by a nurse every two weeks. If necessary, the measurement patterns were adjusted for the patient's convenience. In case patients did not upload the requested data, a message was sent to them through the App.”

Section Description of the comprehensive monitoring system:

“Technical assistance in case of sensor/system failure is provided through a call center.”

**5-xi) Report any prompts/reminders used**

Subjects included in the trial received reminder messages about medication adherence if validation dropped below 80%, or if they did not enter data for two weeks, or if any of the warning lights turned yellow or red according to specific algorithms for either variable.

**5-xii) Describe any co-interventions (incl. training/support)**

“Control and experimental rehabilitation procedures:

Both groups underwent the same educational sessions. During follow-up, all patients were instructed to engage in moderate physical activity at least 150 minutes a week guided by the Borg’s rating of perceived exertion scale of 12-14 (6-20 scale), as well as strength exercises twice a week.”

As already mentioned, control group performed supervised exercise during 16 in-hospital sessions. Physical activity consisted of a workout routine and aerobic cycling training. Warm-up and stretching exercises were included.

Telerehabilitation group performed 4 in-hospital sessions. Physical activity consisted of walking down a corridor, adjusting their pace to the target heart rate (60-80% of heart rate reserve based on baseline treadmill test). Warm-up, stretching and resistance band exercises were also included. The smartphone app guided them through a daily schedule for 10 months. All entered data were saved on the web platform.

**6a) CONSORT: Completely defined pre-specified primary and secondary outcome measures, including how and when they were assessed**

“Outcome measures

Primary outcome was increase in self-reported physical activity in MET-min/week derived from the IPAQ questionnaire at 10 months. The main secondary outcome was increase in VO2max. Additional secondary outcomes included other CPET measurements, changes in laboratory parameters, anthropometric variables, adherence to the rehabilitation programme, returning to work, adherence to a Mediterranean diet, psychological well-being, health-related quality of life and smoking cessation.

Cardiopulmonary stress test

A symptom-limited CPET was performed after hospital discharge and at 10 months. Final CPET required a negative COVID-19 PCR test due to hospital regulations. The stress testing was based on a Bruce protocol using an ergospirometer (Jaeger, MS-CPX, Germany) and a treadmill (T-2100, GE Healthcare). Heart rate, blood pressure, 12-lead ECG, and breath-by-breath respiratory gas analysis were recorded. The test was assumed to be maximal in case of a respiratory gas exchange ratio (RER) >1.1 or cardiac heart rate >85% of maximal predicted heart rate. VO2 was defined as the maximal oxygen uptake during the final 30 seconds of the test. The final CPET was performed with or without beta-blockers depending on their use at baseline.

Blood tests and lipid measurements

Blood samples were drawn at baseline, 4 and 10 months of follow up, to test glucose, glycosylated haemoglobin, creatinine, lipid profile, apolipoproteins and Lp(a), which were subsequently analysed in the hospital central laboratory. No modification of the lipid-lowering therapy was allowed during the study unless the LDL cholesterol was above 100 mg/dL at four months, in which case the treatment was modified and the patient was excluded from the lipid substudy.

Body composition and waist circumference

Measurements at baseline, 4 and 10 months, including weight, visceral fat, BMI, and energy expenditure, were assessed with a Tanita BC-602 scale. Waist circumference was measured midway between the costal border and the iliac crest.

Questionnaires

All patients answered questionnaires at baseline and 10 months. Due to small sample size, questionnaires were administered through computer assisted face-to-face interviewing, in order to obtain higher response rates. The interviewer didn´t know patient assignation. The International Physical Activity Questionnaire (IPAQ) consists of 7 questions about physical activity (intense, moderate, or walking) in the last 7 days, as well as the time spent sitting on a weekday ^14^. The MET values assigned to each level of physical activity are: 3.3 for walking, 4 for moderate physical activity, and 8 for vigorous activity. The MET-minute/week are the result of multiplying each value (3.3, 4 or 8) by the duration in minutes of the daily activity and by the number of days per week that it is performed.

The Prevention with Mediterranean Diet (PREDIMED) questionnaire includes 14 items on adherence to the Mediterranean diet pattern. A score of less than 7 reflects low adherence to the diet, and a score of more than 9 reflects good adherence^15^.

The Hospital Anxiety and Depression Scale (HADS) consist of 14 items divided into anxiety and depression subscales, with 7 items each. The reference period is the previous week. The total score would detect a global affective disorder^16^.

The EQ-5D-5L questionnaire has two parts. The first part measures 5 aspects of health (mobility, self-care, activities of daily life, pain/discomfort, and anxiety/depression) with 5 levels of severity. The second part is a scale from 0 (worst state of health) to 100 (best state of health). An index of reference values is obtained for each health condition. This index can be used to calculate quality-adjusted life years (QALYs): an indicator of the treatments outcomes or, if costs are additionally calculated, of cost-effectiveness or cost-utility studies^17^.

**6a-i) Online questionnaires: describe if they were validated for online use and apply CHERRIES items to describe how the questionnaires**

**were designed/deployed.**

We used computer-assisted offline questionnaires, except for the EQ-5D questionnaire which was administered on paper sheets.

**6a-ii) Describe whether and how “use” (including intensity of use/dosage) was defined/measured/monitored**

“Users' experience with the RC Cardiac Rehabilitation App:

The inclusion rate in the app of requested data was 47% for exercise sessions, 59% for food intake, and 54% for treatment validation. Data entering was considered poor (less than 20%) in 7 patients.”

**6a-iii) Describe whether, how, and when qualitative feedback from participants was obtained**

“Users' experience with the RC Cardiac Rehabilitation App

App user experience of the first 20 participants was assessed with the System Usability Scale. The overall score was 80.4 out of 100”.

“The users' main complaints were Internet connection problems, especially with data transmission at the end of exercise sessions, and handling difficulties in older patients”. No feedback from participants in the control group was obtained”.

**6b) CONSORT: Any changes to trial outcomes after the trial commenced, with reasons**

Study design:

…”Before recruitment, two amendments were accepted: a third group without cardiac rehabilitation was eliminated as it was not considered ethical nor necessary, and maximal oxygen uptake (VO2max) was included as the main secondary outcome”.

The reason for the second amendment was simply that we finally get an ergospirometer available. This fact did not change the initial design of considering physical activity as the primary objective as determined by the IPAQ questionnaire that served in the first instance for the sample size calculation.

No changes to trial outcomes were made after the trial commenced.

**7a) CONSORT: How sample size was determined**

**7a-i) Describe whether and how expected attrition was taken into account when calculating the sample size**

“Sample size

Based on the potential impact of the intervention on compliance with physical activity recommendations, a greater increase in total MET-min/week derived from the IPAQ survey in the experimental group was considered plausible. This gave rise to expected mean values 25 % higher at the end of rehabilitation for the experimental group (5000 MET-min/week versus 4000 for the CBCR group) (standard deviation in both groups was considered equal to 1000). ^19,20^. A total of 30 patients in each group was calculated to provide 95 % power at the 5 % level of significance to detect a statistically significant difference between groups using the Mann-Whitney U test. A 12 % loss to follow-up was estimated; therefore, 70 patients were enrolled in the study.

**7b) CONSORT: When applicable, explanation of any interim analyses and stopping guidelines**

Not applicable.

**8a) CONSORT: Method used to generate the random allocation sequence**

“Study design:

…Patients were randomly assigned (1:1) to either CTR or CBCR regardless of the hospital of origin. Randomisation was done using a permuted block scheme (block size of ten). Previously, 7 blocks of size 10 were randomly selected from patient assignments to both treatment arms”. **8b) CONSORT: Type of randomization; details of any restriction (such as blocking and block size)**

“Study design:

…Randomisation was done using a permuted block scheme (block size of ten). Previously, 7 blocks of size 10 were randomly selected from patient assignments to both treatment arms This selection was carried out by an independent statistician who monitored the random assignment system”.

**9) CONSORT: Mechanism used to implement the random allocation sequence (such as sequentially numbered containers), describing any steps taken to conceal the sequence until interventions were assigned**

“Study design:

The assignments were then stored in seven packages of 10 closed envelopes, each containing the assignment to a treatment. These packages were handed over to the principal investigator, who could only open them after the recruitment of a new patient”.

**10) CONSORT: Who generated the random allocation sequence, who enrolled participants, and who assigned participants to interventions**

Study design:

…”The random allocation sequence was generated by an independent statistician who monitored the random assignment system”.

Participants were enrolled and assigned to interventions by the main investigator.

**11a) CONSORT: Blinding - If done, who was blinded after assignment to interventions (for example, participants, care providers, those assessing outcomes) and how**

**11a-i) Specify who was blinded, and who wasn’t**

“Study design:

Patients were notified of their group allocation in a sealed envelope after the baseline exercise test and asked not to disclose it to the faculty”.

“Participants were not blinded to group assignment due to the nature of each intervention.

The investigators involved in questionnaire administration and CPET evaluation were unaware of the patients' allocation”.

**11a-ii) Discuss e.g., whether participants knew which intervention was the “intervention of interest” and which one was the “comparator”**

When they were asked to participate in the study, it was told that two different ways of performing cardiac rehabilitation would be compared. One was experimental by using a mobile phone app and reduced hospital sessions, and the other was the way it is done in standard rehabilitation units and that to date, both had been shown to be equally useful and effective.

At this point I must admit that most would ideally opt for the group of reduced hospital stays and use of this technology.

**11b) CONSORT: If relevant, description of the similarity of interventions**

The two interventions have many similarities in terms of their intended purpose but are very different in terms of adherence to the recommendations to change lifestyle habits after an acute coronary syndrome.

**12a) CONSORT: Statistical methods used to compare groups for primary and secondary outcomes**

“Statistical analysis

The outcomes were analysed according to the intention-to-treat principle. To analyze treatment effects within groups (at four months or at the end of rehabilitation), we used McNemar-Bowker test of symmetry for qualitative variables (McNemar test for dichotomous variable) or Student t-test for paired samples for quantitative variables (Wilcoxon signed-rank test when parametric assumptions couldn’t be assumed). The comparison of treatments between groups was carried out using Pearson χ2 test for qualitative variables (Fisher exact test for dichotomous variable) or Student t-test for independent samples for quantitative variables (Mann-Whitney U test when parametric assumptions couldn’t be assumed). The relationship between two variables was assessed by Pearson’s correlation coefficient (Spearman’s rank correlation coefficient when parametric assumptions couldn’t be assumed). Two-sided exact p-values were calculated whenever possible and p-values ≤ 0.05 were considered statistically significant. Data were analyzed using IBM SPSS Statistics 22 and R 4.0.2 for Microsoft Windows”.

**12a-i) Imputation techniques to deal with attrition / missing values**

No data imputation was performed for missing values. All data available were considered

**12b) CONSORT: Methods for additional analyses, such as subgroup analyses and adjusted analyses**

Not applicable

RESULTS

**13a) CONSORT: For each group, the numbers of participants who were randomly assigned, received intended treatment, and were analysed for the primary outcome**

34 patients were randomized to the control group. 30 patients underwent the conventional cardiac rehabilitation programme. 28 patients were analysed for the primary endpoint .

33 patients were randomized to the intervention group.

32 patients underwent the cardiac telerehabilitation programme. 30 patients were analysed for the primary endpoint of the study.

**13b) CONSORT: For each group, losses and exclusions after randomisation, together with reasons**

The flow chart include this information as Figure 1.

**13b-i) Attrition diagram**

The flow chart include this information as Figure 1.

**14a) CONSORT: Dates defining the periods of recruitment and follow-up**

“Study population:

Patients were recruited after an acute coronary syndrome (unstable angina, NSTEACS or NSTEACS) between 28 May 2019 and 10 March 2020”.

The follow-up period was 10 months.

**14a-i) Indicate if critical “secular events” fell into the study period**

No secular events fell into the study period.

**14b) CONSORT: Why the trial ended or was stopped (early)**

the study was completed on schedule, only the recruitment period was stopped early due to the

COVID-19 pandemic.

**15) CONSORT: A table showing baseline demographic and clinical characteristics for each group**

This information is provided in Supplementary material 3.

**15-i) Report demographics associated with digital divide issues**

Baseline characteristics of the population include subject age. An inclusion criterion was age between 18 and 72 years. The maximum age of 72 years was arbitrarily chosen to avoid lack of digital tool handling. Patients had to ensure: “minimum smartphone usage skills” as it is mentioned in the study population section.

**16a) CONSORT: For each group, number of participants (denominator) included in each analysis and whether the analysis was by original assigned groups**

This information is included in the paper.

**16-i) Report multiple “denominators” and provide definitions**

This information is included in the paper.

**16-ii) Primary analysis should be intent-to-treat**

“Statistical analysis

The outcomes were analysed according to the intention-to-treat principle”.

**17a) CONSORT: For each primary and secondary outcome, results for each group, and the estimated effect size and its precision (such as 95% confidence interval).**

Primary outcome

“The CTR group reported a significant increase in MET-min/week from baseline, with a median improvement of 1723 units (IQR 697-3295, p=0.005), by increasing walking time, time (median improvement of 446 units, IQR 165-1155, p<0.001), moderate activity, (median improvement of 360 units, IQR 0-1400, p=0.033), and nearly significant vigorous physical activity, activity (median improvement of 360 units, IQR 0-2800, p=0.053). No significant increase was observed in the CBCR group. (Table 1). Compared to control group, increase in total physical activity was significantly higher in the CTR group (median increase of 1726 METS-min/week *vs*. 636 for CBCR group, p=0.045) (Figure 2). The correlation between MET-min/week and VO2max before and after rehabilitation was ρ = 0.224 (p=0.091) and 0.311 (p=0.018) respectively.”

Seconday outcomes

Cardiopulmonary exercise testing

In the CTR group, VO2max mean increase from baseline was 1.7 ml/kg/min (95%CI 0.56-2.69, p<0.004), and in the CBCR group was 0.6 ml/kg/min (p=0.39) (Figure 2). Mean maximal heart rate increase in the CTR group from baseline was 8.5 BPM (95%CI 4.38-12.52, p<0.001), compared to 3.5 BPM in the control group (p=0.18). Exercise time in the CTR group increased 1.45 min. (95%CI 0.96-1.94, p<0.001), compared to 0.49 min in the control group (p=0.27).

Compared to control group, increase in treadmill speed (p=0.019) and elevation (p=0.006), and a nearly significant difference in exercise time (p=0.055), in favor of the CTR group, was observed. (Table 1).

Blood test parameters:

ApoB/ApoA-I ratio decreased significantly in the CTR group with a mean decline of 0.13 (95%CI 0.03-0.24, p=0.017), whereas it did not significantly change in CBCR group (p=0.092). Non-HDL cholesterol increased significantly in the CBCR group with a median increase of 7.3 mg/dL (IQR -2.4-18.55, p=0.021), but did not significantly change in the CTR group (p=0.080). LDL cholesterol increased significantly in both groups, with a median increase of 4.5 mg/dL (IQR=-1-19, p=0.047) in the CTR group and 3.5 mg/dL (IQR=-1.5-15, p=0.040) in the CBCR group. Total cholesterol increased significantly in the CBCR group with a median increase of 11.5 mg/dL (IQR=-4-18.5, p=0.012), whereas it did not significantly change in CTR group (p=0.141). No significant differences between groups on lipid parameters were found (Figure 3). (For all blood test parameters considered, see supplementary material 4)

Questionnaires:

As secondary outcomes derived from de IPAQ questionnaire, the percentage of patients who declared a high level of effort at the end of the study period, was significantly higher in the CTR group compared to the CBCR group (76.7% vs. 42.9%, p=0.031) (Table 1).

The HADS global score improved from baseline significantly in both groups, with a median descent of 6 points in CTR group (IQR=3-11, p<0.001) and 4 points in CBCR group (IQR=0-6, p=0.045), being the improvement significantly greater in CTR group (p=0.015). The anxiety subscale improved significantly in both groups (p<0.001 for CTR group and p=0.010 for CBCR group) with significantly greater effect in CTR group (p=0.006), whereas depression subscale only improved significantly in CTR group (p=0.020).

The PREDIMED score improved from baseline significantly in both groups, with a median improvement of 3 points in CTR group (IQR=2-6, p<0.001) and 2.5 points in CBCR group (IQR=0.5-4, p<0.001), with no differences between groups (p=0.345). The percentage of patients reporting high adherence to the Mediterranean diet (score >9 points) was higher in the CTR group (70%) compared to the CBCR group (32%), p=0.001.

The global EQ-5D-5L questionnaire index increased from baseline significantly in CTR group, with a median improvement of 0.06 points (IQR=0.01-0.11, p=0.003) and 0 points in CBCR group (IQR=0-0.14, p=0.064), with no differences between groups (p=0.261). The self assessment of health improved in both groups but was only significant in the CTR group (p=0.008) (Suplementary material 5).

**17a-i) Presentation of process outcomes such as metrics of use and intensity of use**

In terms of metrics of use, as already mentioned, “it was 47% for exercise sessions, 59% for food intake, and 54% for treatment validation. Data entering was considered poor (less than 20%) in 7 patients.”

**17b) CONSORT: For binary outcomes, presentation of both absolute and relative effect sizes is recommended**

Effect sizes were reported in the paper.

**18) CONSORT: Results of any other analyses performed, including subgroup analyses and adjusted analyses, distinguishing pre-specified**

**from exploratory**

No other analyses were carried out

**18-i) Subgroup analysis of comparing only users**

No other analyses were carried out within the intervention group.

**19) CONSORT: All important harms or unintended effects in each group**

“Patients and programme adherence:

…During the study period, 3 patients were readmitted. Two patients in the CTR group with unstable angina had distal lesions in the stented vessel, not amenable for revascularization. One of them was finally excluded due to refractory angina. In the CBCR group, one patient with post-infarction angina received stenting in a vessel other than the infarct-related, and remained asymptomatic afterwards”.

DISCUSION:

“The longest time to starting rehabilitation in the CBCR group was partly due to unforeseen delay in some patients in the scheduled visit to a rehabilitation physician. It’s not excluded that it may have led to higher dropout in the CBCR group”

**19-i) Include privacy breaches, technical problems**

“Users’ experience with the RC Cardiac Rehabilitation App:

…The users' main complaints were Internet connection problems, especially with data transmission at the end of exercise sessions, and handling difficulties in older patients”.

**19-ii) Include qualitative feedback from participants or observations from staff/researchers**

“Users' experience with the RC Cardiac Rehabilitation App

…App user experience of the first 20 participants was assessed with the System Usability Scale. The overall score was 80.4 out of 100”.

The design of this system has been a labour-intensive process. It was inspired by the INTERHEART study. The result is good as it includes the main prognostic variables that determine the appearance of an acute coronary event. In our opinion, it includes all that a Cardiac Rehabilitation or Secondary Prevention Unit may need for an optimal follow-up of patients.

**20) CONSORT: Trial limitations, addressing sources of potential bias, imprecision, multiplicity of analyses**

**20-i) Typical limitations in eHealth trials**

Bias in the results caused by the non-use of tele-intervention was not ruled out.

**21) CONSORT: Generalizability (external validity, applicability) of the trial findings**

**21-i) Generalizability to other populations**

DISCUSSION:

…” It appears to be even a more effective alternative to conventional rehabilitation at least in low-risk patients, as these results support. This new scheme, which redefines the concepts of phase II and III of cardiac rehabilitation, could achieve the hypothetical objective of a 80% inclusion rate of eligible patients, ensuring at the same time early return to daily life and making efficient use of health resources.

If the results were confirmed by a larger study, the use of digital tools, such as the one proposed, could lead to a change in the standard-of-care model for cardiac rehabilitation in low-to-moderate risk patients in the near future.”

Current recommendations of Scientific Societies recommend the use of telerehabilitation. As has been shown in other studies, this strategy of prolonged follow-up by telemonitoring could be extended to patients at moderate risk. This is our next goal. It has not been tested in patients with heart failure, although there are previous experiences of safety and efficacy that are comparable to hospital programmes.

**21-ii) Discuss if there were elements in the RCT that would be different in a routine application setting**

The origin of this technological development arises from the search for alternatives to the lack of space and equipment in our hospital to start a cardiac rehabilitation programme. Once this tool has been validated, only cardiac rehabilitation will be carried out in our centre using this technology, with the minimum hospital stay of one or two sessions and using a corridor as the only facility. Therefore, the answer is that it can be used in any hospital.

It is very useful to have an external call centre to solve the frequent queries about the system operation and data transfer failures.

**22) CONSORT: Interpretation consistent with results, balancing benefits and harms, and considering other relevant evidence**

**22-i) Restate study questions and summarize the answers suggested by the data, starting with primary outcomes and process outcomes (use)**

DISCUSSION:

“Our trial shows that a 10-month programme of CTR, without a traditional in-hospital phase II, increases physical activity, oxygen consumption, improves the lipid risk profile, quality of life, and encourages adherence to the programme, compared to a CBCR programme”.

“Our primary outcome shows significant increase in self-reported physical activity, consistent with previous data. This benefit might be related to the longer duration or to the higher adherence of the programme”

“Among the blood test results, are noteworthy the decrease in ApoB/ApoA-I ratio in the CTR group and the significant increase in non-HDL cholesterol in the control group”

“We found a significant reduction in the return to work lapse in the CTR group”

“Several surveys during the COVID-19 pandemic have suggested a higher prevalence of anxiety, depression and lower well-being compared with historical estimates. The benefit observed in the CTR group, especially in the depression subscale, it is noticeable”

**22-ii) Highlight unanswered new questions, suggest future research**

When asked whether these observed differences could be prolonged over time, all patients were asked to repeat the questionnaires, and CPET at 20 months, and most of them agreed to continue. We therefore hope to have these results.

This intervention could be feasible in patients with moderate risk, more doubtfully in patients with severe risk. Also, in patients recently discharge with heart failure, for which we are adapting the system.

Other information

**23) CONSORT: Registration number and name of trial registry**

The study protocol was included in the Clinical Trials.gov. Identifier: NCT 04942977

**24) CONSORT: Where the full trial protocol can be accessed, if available**

The full trial protocol can be accessed as supplementary file (Supplementary material 1).

**25) CONSORT: Sources of funding and other support (such as supply of drugs), role of funders**

Funding for the clinical trial has been provided by the Trilema Foundation.

[www.fundaciontrilema.org](http://www.fundaciontrilema.org/)

[www.movisalud.es/en/](http://www.movisalud.es/en/)

**X26-i) Comment on ethics committee approval**

“Study design:

The trial was conducted with the ethical principles contained in the Declaration of Helsinki. The study was approved by the local ethics committee and the Spanish Agency of Medicines and Medical Devices”

The protocol's approval by the ethics committee is included in the minutes 2/2014 dated 29 January 2014.

**x26-ii) Outline informed consent procedures**

Study design:

“Each participant signed an informed consent prior to participation”.

As stated in the clinical trial protocol:

The study investigator will explain to patients (or their legally authorised representatives) the nature of the study, its purposes, procedures, estimated duration, potential risks and expected benefits. It will be clearly stated that participation is voluntary and that they may withdraw from the study at any time and without detriment to their future health care. Before signing, you should read the contents and comment or ask any questions you may have. Two documents shall be signed and kept by the patient and the investigator. No procedure will be carried out unless this consent form has been signed.

**X26-iii) Safety and security procedures**

As stated in the clinical trial protocol:

The confidentiality of each patient's data will be respected at all times. The affiliation of each of the patients and their association with the study and, thus, access to the data collected in the CRF is ensured by a document to which only the investigators, monitors and health authorities, if applicable, have access. The CRFs contain only the identification number of each subject included. The data obtained will be handled in accordance with the Spanish regulations on the handling of computerized data. (LO 15/1999, of 13 December on the protection of personal data).

**X27-i) State the relation of the study team towards the system being evaluated**

The authors declare that they have no conflict of interest.

The main investigator worked on the design and fine-tuning of the system. He has not received any economic compensation for this dedication.
