# Supplementary material for: A randomized controlled clinical trial of cardiac telerehabilitation with a prolonged mobile care monitoring strategy after an acute coronary syndrome
Source: Clin Cardiol. 2021 Dec 24;45(1):31–41. doi: 10.1002/clc.23757 (PMC8799046; doi:10.1002/clc.23757)
Supplement: Supplementary file 3 — Supporting information. [file CLC-45-31-s004.docx]

**Supplementary material 3.**

|  | CTR group | | | CBCR group | | | p_12_ |  |  | p |
| --- | --- | --- | --- | --- | --- | --- | --- | --- | --- | --- |
|  | Baseline | Final | p_1_ | Baseline | Final | p_2_ |  |  |  |  |
| **IPAQ** | (n=30) | | | (n=28) | | |  |  |  |  |
| Total MET-min/week, m(IQR) | 1251 (693-2624) | 4031 (1875-5973) | 0.005 | 1502 (896-3924) | 2420 (1391-4997) | 0.374 | 0.045 |  |  |  |
| Walking, m(IQR) | 743 (231-1386) | 1287 (990-2079) | <0.001 | 644 (330-1386) | 1172 (743-1386) | 0.070 | 0.350 |  |  |  |
| Moderate activity, m(IQR) | 140 (0-720) | 580 (240-2400) | 0.033 | 440 (0-2700) | 540 (0-1020) | 0.548 | 0.039 |  |  |  |
| Vigorous activity, m(IQR) | 0 (0-320) | 620 (0-2400) | 0.053 | 0 (0-240) | 0 (0-1960) | 0.313 | 0.484 |  |  |  |
| Hours a day sitting, m(IQR) | 7 (5-8) | 6 (4-7) | 0.147 | 6 (5-9) | 7 (6-8) | >0.800 | 0.215 |  |  |  |
| Weekly energy expend., m(IQR) | 1524 (773-3892) | 5445 (2905-8772) | 0.002 | 2207 (1334-8505) | 3349 (1956-7491) | 0.767 | 0.036 |  |  |  |
| Effort level |  |  | <0.001 |  |  | 0.343 | 0.031 |  |  |  |
| Inactive/low, n (%) | 6 (20.0%) | 1 (3.3%) |  | 6 (21.4%) | 3 (10.7%) |  |  |  |  |  |
| Moderate, n (%) | 17 (56.7%) | 6 (20.0%) |  | 12 (42.9%) | 13 (46.4%) |  |  |  |  |  |
| High, n (%) | 7 (23.3%) | 23 (76.7%) |  | 10 (35.7%) | 12 (42.9%) |  |  |  |  |  |
| **HADS** | (n=27) | | | (n=27) | | |  |  |  |  |
| Total score, m(IQR) | 9 (6-14) | 2 (1-6) | <0.001 | 6 (4-11) | 2 (1-9) | 0.045 | 0.015 |  |  |  |
| Anxiety, m(IQR) | 6 (3-9) | 1 (0-1) | <0.001 | 4 (2-6) | 1 (0-4) | 0.010 | 0.006 |  |  |  |
| Absence (0-7), n (%) | 18 (66.7%) | 27 (100.0%) |  | 22 (81.5%) | 25 (92.6%) |  |  |  |  |  |
| Moderate (8-10), n (%) | 6 (22.2%) | 0 (0.0%) |  | 4 (14.8%) | 1 (3.7%) |  |  |  |  |  |
| Relevant (11-21), n (%) | 3 (11.1%) | 0 (0.0%) |  | 1 (3.7%) | 1 (3.7%) |  |  |  |  |  |
| Depression, m(IQR) | 4 (2-7) | 2 (0-4) | 0.020 | 2 (0-5) | 2 (0-5) | 0.600 | 0.188 |  |  |  |
| Absence (0-7), n (%) | 24 (88.9%) | 25 (92.6%) |  | 23 (85.2%) | 25 (92.6%) |  |  |  |  |  |
| Moderate (8-10), n (%) | 2 (7.4%) | 1 (3.7%) |  | 3 (11.1%) | 1 (3.7%) |  |  |  |  |  |
| Relevant (11-21), n (%) | 1 (3.7%) | 1 (3.7%) |  | 1 (3.7%) | 1 (3.7%) |  |  |  |  |  |
| **PREDIMED** | (n=30) | | | (n=28) | | |  |  |  |  |
| Total score, m(IQR) | 6 (6-8) | 11 (9-12) | <0.001 | 6 (4-8) | 8 (6-11) | <0.001 | 0.345 |  |  |  |
| Adherence |  |  | <0.001 |  |  | 0.014 | 0.001 |  |  |  |
| Low (<7), n (%) | 16 (53.3%) | 0 (0.0%) |  | 15 (53.6%) | 9 (32.1%) |  |  |  |  |  |
| Intermediate (7-8), n (%) | 9 (30.0%) | 9 (30.0%) |  | 12 (42.9%) | 10 (35.7%) |  |  |  |  |  |
| High (>9) (%), n (%) | 5 (16.7%) | 21 (70.0%) |  | 1 (3.6%) | 9 (32.1%) |  |  |  |  |  |
| **EQ-5D-5L** | (n=29) | | | (n=28) | | |  |  |  |  |
| Index, m(IQR) | 0.86 (0.82-0.93) | 0.93 (0.91-1.00) | 0.003 | 0.91 (0.86-1.00) | 1.00 (0.91-1.00) | 0.064 | 0.261 |  |  |  |
| Analog scale, m(IQR) | 70 (65-85) | 80 (70-90) | 0.008 | 75 (60-90) | 88 (68-90) | 0.064 | >0.800 |  |  |  |
| Mobility, m(IQR) | 1 (1-1) | 1 (1-2) | 0.058 | 1 (1-1) | 1 (1-1) | 0.577 | 0.279 |  |  |  |
| Self-care, m(IQR) | 1 (1-1) | 1 (1-1) | 0.157 | 1 (1-1) | 1 (1-1) | >0.800 | 0.161 |  |  |  |
| Daily activities, m(IQR) | 1 (1-1) | 1 (1-1) | 0.008 | 1 (1-1) | 1 (1-1) | 0.546 | 0.162 |  |  |  |
| Pain/Discomfort, m(IQR) | 2 (1-2) | 1 (1-2) | 0.059 | 2 (1-2) | 1 (1-2) | 0.315 | 0.959 |  |  |  |
| Anxiety/Depression, m(IQR) | 1 (1-2) | 1 (1-1) | 0.033 | 1 (1-2) | 1 (1-1) | 0.132 | 0.496 |  |  |  |

m = median, IQR =interquartile range, p_1_ = changes in CTR group, p_2_ = changes in CBCR group. p_12_ = comparison changes between groups.
