# Supplementary material for: A randomized controlled clinical trial of cardiac telerehabilitation with a prolonged mobile care monitoring strategy after an acute coronary syndrome
Source: Clin Cardiol. 2021 Dec 24;45(1):31–41. doi: 10.1002/clc.23757 (PMC8799046; doi:10.1002/clc.23757)
Supplement: Supplementary file 4 — Supporting information. [file CLC-45-31-s002.docx]

**Supplementary material 4.**

|  | CTR group | | | CBCR group | | | p_12_ |
| --- | --- | --- | --- | --- | --- | --- | --- |
|  | Baseline | Final | p_1_ | Baseline | Final | p_2_ |  |
| Glycosilated Hb (%), median (IQR) (n_1_=25, n_2_=25) | 5.8 (5.6-6.1) | 5.8 (5.6-6.2) | 0.763^b^ | 5.7 (5.4-6.0) | 5.7 (5.5-6.1) | 0.209 | 0.305 ^b^ |
| Glucose (mg/dL), median (IQR) (n_1_=30, n_2_=25) | 104 (98-114) | 110 (99-118) | 0.100 ^b^ | 98 (95-108) | 106 (95-120) | 0.117 | >0.8 ^b^ |
| Creatinine (mg/dL), mean(SD) (n_1_=30, n_2_=25) | 0.93 (0.19) | 0.92 (0.19) | 0.417 ^a^ | 1.03 (0.22) | 1.00 (0.21) | 0.150 | 0.484 ^a^ |
| GFR (mL/min/1.73m2) |  |  | >0.8^c^ |  |  | 0.219 | >0.8^c^ |
| <90, n(%) | 16 (59.3%) | 15 (55.6%) |  | 18 (72.0%) | 14 (56.0%) |  |  |
| >=90, n(%) | 11 (40.7%) | 12 (44.4%) |  | 7 (28.0%) | 11 (44.0%) |  |  |
| Cholesterol (mg/dL), median (IQR) (n_1_=30, n_2_=24) | 125 (108-224) | 124 (108-144) | 0.141 ^b^ | 122 (110-137) | 124 (107-155) | 0.012 | 0.607 ^b^ |
| HDL cholesterol (mg/dL), mean(SD) (n_1_=31, n_2_=24) | 46.6 (12.1) | 45.5 (9.2) | 0.421 ^a^ | 45.7 (10.2) | 46.2 (9.4) | >0.8 | 0.545 ^a^ |
| Cholesterol/HDL ratio, median (IQR) (n_1_=30, n_2_=24) | 2.70 (2.35-3.04) | 2.84 (2.43-3.23) | 0.120 ^b^ | 2.55 (2.25-3.05) | 2.80 (2.20-3.47) | 0.137 | >0.8 ^b^ |
| Non-HDL cholesterol, median (IQR) (n_1_=30, n_2_=24) | 76.4 (66.2-90.8) | 83.5 (61.0-99.0) | 0.080 ^b^ | 73.1 (59.3-88.9) | 82.5 (59.0-106.0) | 0.021 | >0.8 ^b^ |
| VLDL cholesterol (mg/dL), median (IQR) (n_1_=29, n_2_=24) | 18 (15-24) | 17 (14-22) | 0.745 ^b^ | 18 (14-25) | 20 (14-31) | 0.738 | 0.654 ^b^ |
| LDL cholesterol (mg/dL), median (IQR) (n_1_=30, n_2_=24) | 59 (44-69) | 60 (47-73) | 0.047 ^b^ | 53 (39-68) | 57 (43-83) | 0.040 | >0.8 ^b^ |
| Triglycerides (mg/dL), median (IQR) (n_1_=29, n_2_=24) | 91 (77-121) | 85 (71-110) | 0.787 ^b^ | 87 (71-123) | 98 (68-153) | 0.648 | 0.520 ^b^ |
| GGT (IU/L), median (IQR) (n_1_=30, n_2_=24) | 32 (21-39) | 26 (22-32) | 0.229 ^b^ | 33 (24-45) | 33 (24-40) | >0.8 | 0.626 ^b^ |
| C-reactive protein (mg/L), median (IQR) (n_1_=28, n_2_=24) | 1.9 (0.6-3.6) | 1.1 (0.6-2.7) | 0.127 ^b^ | 2.2 (1,0-4.1) | 1.2 (0.6-2.6) | 0.021 | 0.741 ^b^ |
| Apolipoprotein A-I (mg/dL), mean(SD) (n_1_=30, n_2_=24) | 130.9 (35.1) | 151.9 (27.2) | <0.001 ^a^ | 131.7 (30.1) | 155.7 (23.3) | <0.001 | 0.665 ^a^ |
| Apolipoprotein B (mg/dL), median (IQR) (n_1_=30, n_2_=24) | 70 (61-89) | 74 (58-88) | >0.8 ^b^ | 72 (56-80) | 78 (53-88) | 0.415 | 0.536 ^b^ |
| ApoB/apoA-I ratio, mean(SD) (n_1_=30, n_2_=24) | 0.62 (0.28) | 0.49 (0.14) | 0.017^a^ | 0.56 (0.20) | 0.48 (0.15) | 0.092 | 0.450^a^ |
| Lipoprotein(a) (mg/dL) median (IQR) (n_1_=30, n_2_=24) | 44 (11-99) | 43 (13-105) | 0.268 ^b^ | 29 (13-62) | 30 (14-70) | 0.747 | 0.513 ^b^ |

*^a^Student’s t-test for paired samples. ^b^Wilcoxon signed-rank test. ^d^ McNemar’s test.* SD = standard deviation, IQR =interquartile range, p1 = changes in CTR group.

P2 = changes in CBCR group. p12 = comparison changes between two groups, n_1_(n_2_) = sample size for effect analysis in CTR (CBCR) group.
